# Breast cancer incidence, by stage at diagnosis, and mortality in 21 European countries in the era of mammography screening: an international population-based study

**DOI:** 10.1016/j.lanepe.2025.101574

**Published:** 2025-12-29

**Authors:** Rafael Cardoso, Idris Ola, Lina Jansen, Monika Hackl, Petra Ihle, Julie Francart, Nancy Van Damme, Zdravka Valerianova, Trayan Atanasov, Ondřej Májek, Ondřej Ngo, Kaire Innos, Margit Mägi, Sandrine Dabakuyo Yonli, Anne-Sophie Woronoff, Brigitte Tretarre, Florence Poncet, Alexander Katalinic, Paul M. Walsh, Ieva Vincerževskienė, Laura Steponaviciene, Linda de Munck, Sabine Siesling, Tom Børge Johannesen, Solveig Hofvind, Rita Calisto, Maria José Bento, Filipa Alves da Costa, Alexandra Mayer, António Lourenço, Tina Žagar, Sonja Tomšič, Miguel Rodríguez-Barranco, Maria-José Sánchez, Arantza Lopez de Munain Marques, Rafael Marcos-Gragera, Arantza Sanvisens, Antonia Sánchez Gil, María-Dolores Chirlaque Lopez, Jaume Galceran, Francina Saladié, Kristina Sundquist, Jan Sundquist, Mats-Åke Persson, Marcel Blum, Mohsen Mousavi, Roger von Moos, Olena Sumkina, Anton Ryzhov, Michael Hoffmeister, Hermann Brenner

**Affiliations:** a Division of Clinical Epidemiology of Early Cancer Detection, German Cancer Research Center (DKFZ), Heidelberg, Germany; b Medical Faculty Heidelberg, University of Heidelberg, 69120, Heidelberg, Germany; c Epidemiological Cancer Registry Baden-Württemberg, German Cancer Research Center (DKFZ), Heidelberg, Germany; d Austrian National Cancer Registry, Statistics Austria, Vienna, Austria; e Belgian Cancer Registry, Brussels, Belgium; f Bulgarian National Cancer Registry, University Hospital of Oncology, Sofia, Bulgaria; g Institute of Health Information and Statistics of the Czech Republic, Prague, Czech Republic; h Institute of Biostatistics and Analyses, Faculty of Medicine, Masaryk University, Brno, Czech Republic; i Department of Epidemiology and Biostatistics, National Institute for Health Development, Tallinn, Estonia; j National Institute for Health Development, Tallinn, Estonia; k Breast Cancers and Gynecological Cancers Registry of Côte d'Or, Dijon, France; l UMR INSERM U1231, University of Burgundy, Dijon, France; m Cancer Registry of Doubs, Centre Hospitalier Régional Universitaire Besançon, Besançon, France; n Hérault Tumor Registry, Montpellier, France; o Cancer Registry of Isère, Grenoble, France; p Cancer Registry of Schleswig-Holstein, Lübeck, Germany; q National Cancer Registry Ireland, Cork, Ireland; r Clinical Research Center (Clinic), National Cancer Institute, Vilnius, Lithuania; s Department of Research and Development, Netherlands Comprehensive Cancer Organisation (IKNL), Utrecht, the Netherlands; t Department of Health Technology & Services Research, Technical Medical Center, University of Twente, Enschede, the Netherlands; u Cancer Registry of Norway, The Norwegian Institute of Public Health, Oslo, Norway; v Department of Epidemiology, North Region Cancer Registry of Portugal (RORENO), Portuguese Oncology Institute of Porto (IPO Porto), Porto, Portugal; w Group of Epidemiology, Outcomes, Economics and Management in Oncology – Research Center, Porto Comprehensive Cancer Center (Porto.CCC) & RISE@CI-IPOP (Health Research Network), Portugal; x Population Studies Department, School of Medicine and Biomedical Sciences, ICBAS, University of Porto, Porto, Portugal; y Epidemiology Research Unit, South Portugal Cancer Registry (ROR-Sul), Portuguese Oncology Institute of Lisbon (IPO Lisbon), Lisbon, Portugal; z Faculty of Pharmacy, University of Lisbon, Research Institute for Medicines, Portugal; aa Slovenian Cancer Registry, Institute of Oncology, Ljubljana, Slovenia; ab Granada Cancer Registry, Andalusian School of Public Health, Granada, Spain; ac Instituto de Investigación Biosanitaria de Granada (ibs.GRANADA), Granada, Spain; ad Consortium for Biomedical Research in Epidemiology and Public Health (CIBER Epidemiología y Salud Pública, CIBERESP), Madrid, Spain; ae Basque Country Cancer Registry, Vitoria-Gasteiz, Spain; af Epidemiology Unit and Girona Cancer Registry, Oncology Coordination Plan, Department of Health Government of Catalonia, Catalan Institute of Oncology, Josep Carreras Leukaemia Research Institute (IJC), Girona, Spain; ag Descriptive Epidemiology, Genetics and Cancer Prevention Group, Biomedical Research Institute (IDIBGI-CERCA), Salt, Spain; ah Department of Epidemiology, Regional Health Council, IMIB-Arrixaca, Murcia, Spain; ai Department of Epidemiology, Regional Health Council, IMIB-Arrixaca, Murcia University, Murcia, Spain; aj Tarragona Cancer Registry, Epidemiology and Prevention Cancer Service, Hospital Universitari Sant Joan de Reus, Pere Virgili Health Research Institute, Reus, Spain; ak Department of Clinical Sciences, Center for Primary Health Care Research, Lund University, University Clinic Primary Care, Skåne University Hospital, Region Skåne, Malmö, Sweden; al Cancer Registry of Eastern Switzerland and Liechtenstein, St. Gallen, Switzerland; am Graubünden and Glarus Cancer Registry, Chur, Switzerland; an National Cancer Registry of Ukraine, National Institute of Cancer, Kyiv, Ukraine; ao Taras Shevchenko National University of Kyiv, Kyiv, Ukraine; ap Cancer Prevention Graduate School, German Cancer Research Center (DKFZ), Heidelberg, Germany; aq Department of Medical Sciences, Medical School, University of Girona, Girona, Spain

**Keywords:** Breast cancer, Cancer screening, Mammography, Population-based study, Cancer epidemiology

## Abstract

**Background:**

Mammography screening programmes have been widely implemented across European countries over the past 40 years with the main aim to detect breast cancer earlier and thereby reduce breast cancer mortality. This study aimed to analyse and compare changes over time in breast cancer incidence, by stage at diagnosis, and breast cancer mortality across countries in relation to the timing of screening implementation and age at diagnosis.

**Methods:**

In this population-based study conducted in 21 European countries, data from cancer registries covering over 3 million female patients diagnosed with breast cancer, along with data from national statistical offices from 1978 to 2019 were analysed. Annual age-standardised breast cancer incidence rates (by stage and age at diagnosis) and age-standardised breast cancer mortality rates were calculated. Average annual percent changes (AAPCs) in these metrics within 10 years before and 10 years after screening implementation were estimated.

**Findings:**

Overall, breast cancer incidence rates increased over the study period, with the largest increases observed in the first two decades (1978–1987 and 1988–1997), and AAPCs of up to 4.55 (95% confidence interval, CI, 2.56–6.57). In contrast, breast cancer mortality rates decreased most predominantly in the last two decades (1998–2007 and 2008–2018/19), with AAPCs down to −5.40 (95% CI, −9.70 to −0.89). The largest increases in incidence were seen for in situ and stage I cancers (AAPCs ranging from non-significant to 12.03 (95% CI, 7.40–16.86) following screening implementation). Incidence of stage IV cancer declined or remained stable in most countries, with AAPCs down to −6.16 (95% CI, −8.28 to −4.00) after screening introduction. These trends were particularly pronounced among age groups targeted by screening (mostly 50–69 years). Breast cancer mortality rates declined by up to 3 percent annually after screening initiation (lowest AAPC estimate −3.29 (95% CI, −6.26 to −0.23); yet, AAPCs as low as −2.54 (95% CI, −3.15 to −1.93) were also observed before introduction of screening programmes in countries where implementation occurred later, in the 2000s.

**Interpretation:**

This study suggests that mammography screening has influenced trends in breast cancer incidence and mortality in European countries. The results point to the contribution of mammography screening, alongside advances in diagnostics and treatment, to the observed reductions in breast cancer mortality.

**Funding:**

There was no funding source for this study.

**Research in context**

**Evidence before this study**

We searched PubMed for articles published from database inception up to September 30, 2025, reporting on the effects of breast cancer screening programmes as well as time trends in breast cancer incidence and mortality in European countries. We used the following search terms: (“incidence” OR “mortality” OR “screening”) AND “breast cancer” AND “Europe∗”. Evidence on the effectiveness of breast cancer screening has historically relied on randomised controlled trials conducted more than 30–40 years ago, with meta-analyses of these trials reporting an estimated 20% reduction in breast cancer mortality among women invited for screening over 13 years of follow-up. More recent insights have come from observational studies, reflecting real-world outcomes in the context of widespread implementation of mammography screening across Europe. However, these studies often provide national or subnational estimates, while substantial variation exists in screening strategies, including timing, coverage, and uptake across countries.

**Added value of this study**

This study is, to our knowledge, the most comprehensive, population-level evidence analysis of breast cancer incidence and mortality changes in the last three to four decades in Europe, examining incidence and mortality changes for periods before and after the introduction of screening and providing detailed estimates by stage and age at diagnosis. Drawing on data from 21 European countries, we assessed time trends across the various geographical areas in Europe, and various levels of screening roll out.

**Implications of all the available evidence**

Overall, our findings suggest that mammography screening programmes have contributed to increased detection of in situ and stage I cancer alongside decreases in detection of advanced stage (particularly stage IV) cancer, thereby playing a role in the observed declines in breast cancer mortality rates. Nevertheless, we observed large heterogeneity of trends across European countries that point to varying effects of screening and call for further research and policies to improve breast cancer screening and outcomes in the region.

## Introduction

Breast cancer was estimated to be the most commonly diagnosed cancer and the leading cause of cancer death among females in Europe in 2022, with about 470,000 new cases and 120,000 deaths.^1^

Major drivers of the high incidence (and mortality) rates in Europe include reproductive factors (fewer children and advanced age at first birth), hormonal factors (use of menopausal hormone-replacement therapy and oral contraceptives), and behavioural factors (e.g., alcohol consumption and excess body weight).^2^, ^3^, ^4^, ^5^ Nevertheless, there have also been considerable improvements in quality of care and treatment, as well as in implementation of mammography screening programmes, with potential to improve survival and reduce breast cancer mortality.^6^, ^7^, ^8^

The widespread implementation of mammography screening programmes in European countries over the past four decades was driven by evidence from randomised controlled trials and observational studies suggesting that women invited to mammography screening had a 20% lower risk of dying from breast cancer as compared to those not offered screening.^9^ The screening strategies vary, however, considerably across countries, particularly in timing of implementation, coverage, and uptake.^10^ Also, in some countries (in our study, Bulgaria and Ukraine), organised population-based programmes have not yet been implemented or only pilot programmes are available. Such heterogeneity is likely to have contributed considerably to differences in breast cancer incidence and mortality rates across European countries.

In this study, we provide a comprehensive analysis of breast cancer incidence, by stage at diagnosis, and breast cancer mortality (by analysing changes over the study period and before and after screening implementation) in European countries since the late 1970s, when the first population-based mammography screening programmes started to be rolled out. The emphasis was on comparative analyses between countries according to timing of screening implementation and between various age groups (targeted and not targeted by screening).

This multi-country analysis therefore seeks to illuminate understanding of temporal trends and patterns in breast cancer burden among females over the past decades across European countries, and to provide insights that can guide screening programmes in Europe and beyond.

## Methods

### Study design and data sources

In this population-based study, breast cancer incidence and breast cancer mortality rates were calculated for the time period 1978–2019, and changes in these metrics for time periods before and after mammography screening implementation, in a total of 21 European countries.

Individual-level data from female cases of invasive breast cancer (code C50 in the International Classification of Diseases 10th revision [ICD-10]) were obtained from 34 population-based cancer registries in 18 European countries, and from in situ breast cancer cases (ICD-10 code D05) from 29 registries in 14 countries to analyse incidence rates. Data were obtained for the period 1978–2019, or from the earliest to the latest year within this time window, depending on data availability. 1978, as the beginning of the study period, was chosen as around that time mammography screening programmes started to be initiated in some European countries and population-based cancer registry data also started to become available. 2019 was the latest calendar year with available data at the time of analysis and also the year prior to the COVID-19 pandemic.

The data included patient- and tumour-level characteristics, notably date of diagnosis, sex (male/female), age at diagnosis, topography, morphology, vital status at last contact (alive/deceased) and survival time, and clinical and pathological tumour, node, and metastasis (TNM) information according to the Union for International Cancer Control TNM Classification of Malignant Tumours edition in place at the time of diagnosis. Data sources and relevant quality indicators are shown in Supplementary Table S1.

Numbers of deaths from breast cancer and population numbers by calendar year and five-year age groups (same countries/regions and time windows as for the patient-level data, based on data availability) were obtained from the national statistical offices of each country and were also provided by the cancer registries (Supplementary Table S1). These data were used to calculate breast cancer mortality rates.

In addition, breast cancer incidence and mortality rates for three Nordic countries from which individual level data could not be obtained were retrieved from NORDCAN^11^ and added to the analysis. Thus, in total, data from 21 countries were included (16 with nationwide data and 5 with data for some regions) (Table 1).

**Table 1:** Number of invasive and in situ breast cancer cases included in analyses of incidence by country/region.

Country/region	Years	Invasive cases included in analyses of incidence (N)	Invasive cases with unclassified or unknown stage		In situ cases (N)
			N	%	
**Austria**	1983–2018	169,404	29,492	17.4	11,423
**Belgium**					
Flanders	2001–2018	105,376	7987	6.6	12,020
Wallonia/Brussels	2004–2018	64,615	6369	9.9	7219
**Bulgaria**	1993–2013	72,781	2909	4.0	Not available
**Czech Republic**	1980–2018	189,875	14,709	7.7	9360
**Denmark**^a^	1978–2019	154,073	Not available		Not available
**England**^b^	1995–2019	993,319	42,110	11.5	55,862
**Estonia**	1995–2018	15,601	1149	7.4	469
**Finland**^a^	1978–2019	137,458	Not available		Not available
**France**					
Cote d’Or^c^	1982–2018	12,303	186	1.5	1543
Doubs^d^	1978–2018	12,091	618	9.1	757^d^
Herault	1994–2018	20,257	238	1.2	3286
Isere^d^	1979–2018	28,148	2313	14.7	2542
**Germany (6 states)**^e^	2003–2018	291,093	92,707	31.8	23,347
Germany (Bavaria)	2002–2018	171,900	49,305	28.7	12,678
Germany (Bremen)	1999–2018	11,892	3464	29.1	910
**Iceland**^a^	1978–2019	6203	Not available		Not available
**Ireland**	1994–2017	56,731	4812	8.5	5533
**Lithuania**	1978–2016	44,105	3308	7.5	Not available
**Netherlands**	1989–2018	350,387	4900	1.4	42,760
**Norway**^f^	1978–2019	102,090	12,327	19.1	5903
**Portugal**					
North	2000–2015	27,661	8081	29.2	Not available
South	1998–2018	64,696	12,954	20.0	Not available
**Slovenia**^g^	1978–2018	37,698	859	4.3	1925
**Spain**					
Andalucia	2002–2016	6518	837	12.8	435
Basque Country^h^	1986–2016	35,127	1379	12.8	1261
Girona^i^	1980–2018	11,788	1170	12.3	969
Murcia^j^	1983–2015	15,842	1966	15.4	725
Tarragona^k^	1985–2015	10,412	1219	25.1	351
**Switzerland**					
St. Gallen^l^	1980–2019	9727	68	1.3	524
Grisons	1989–2019	3655	162	4.4	297
**Sweden**^m^	1978–2018	232,001	18,401	17.1	9705
**Ukraine**^n^	2000–2018	253,809	6453	2.5	Not available

a For Denmark, Finland and Iceland, aggregated data were obtained from NORDCAN. Data on in situ cancers and by stage were not available.

b For England, data on stage were only available from 2012 on.

c For France (Cote d’Or), there were 8 cases with unknown age at diagnosis that were not considered in the analysis.

d For France (Doubs and Isere), data on stage were only available from 2002 on.

e The six states for which data were available are Schleswig–Holstein, Hamburg, Lower Saxony, North Rhine Westphalia (administrative district Muenster), Rhineland Palatinate, and Saarland.

f For Norway, data on stage were only available from 1998 on.

g For Slovenia, data on stage were only available from 2003 on.

h For Spain (Basque Country), data on stage were only available from 2010 on.

i For Spain (Girona), data on stage only available from 1993 on.

j For Spain (Murcia), data on stage only available from 1994 on.

k For Spain (Tarragona), there were 6 cases with unknown age at diagnosis that were not considered in the analysis. Also, data on stage were only available from 2005 on.

l For Switzerland (St. Gallen), there were 5 invasive cases with unknown year of and age at diagnosis which were not considered in the analysis. Also, data on stage were only available from 2003 on.

m For Sweden, data on stage were only available from 2004 on.

n For Ukraine, data from the regions Donetsk, Luhansk and Crimea were not included.

In order to analyse incidence and mortality rates in relation to breast cancer screening implementation, relevant characteristics and parameters of breast cancer screening programmes in all included countries, namely timing of introduction, target age groups, screening intervals, and utilisation were also summarised herein (Supplementary Table S2). These were retrieved from a recent comprehensive analysis of the status of breast cancer screening across European countries.^10^

Given the large number of countries/regions included in the analyses and differences in timing of screening implementation, we grouped countries into: i) those with introduction of screening before 1998, i.e., by the end of the first half of the investigation period; ii) after 1998; and iii) without any screening programme or only small-scale pilot programmes during the study period.

This study was approved by the Ethics Committee of the Medical Faculty of Heidelberg University (S-473/2021).

### Statistical analysis

Age-standardised incidence (including stage-specific incidence) and mortality rates (per 100,000 persons) were calculated for each country/region and for each year using the 1976 European Standard Population. This standard population has been widely used in other Europe-wide studies.^12^ Analyses by age (age groups 0–49, 50–69, 70–79, and ≥80 years; age-standardised within each group) were also done for countries from which nationwide data were available and Bavaria, Germany, due to its large population size.

Stages I, II, III, and IV were derived from the clinical and pathological TNM information at diagnosis. Stage information was unknown for 1.2%–31.8% of cases across countries (Table 1). As the proportion of cases with unknown stage changed over time across registries, multiple imputation by chained equations (15 imputed datasets were created using 10 iterations) was done for each registry dataset individually. The imputation model included the following factors: year of diagnosis; age at diagnosis; stage at diagnosis; topography; vital status; the Nelson-Aalen estimator; and region/federal state (Belgium and Germany).

Data on all other variables were available for all cases unless otherwise specified under Table 1 (only very small numbers with missing data for some countries).

Average annual percent changes (AAPCs) in incidence, stage-specific incidence, and mortality were estimated for the 10-year period preceding screening implementation and for the 10-year period starting from the year prior to screening introduction for each country/region. These time periods were chosen to be able to include data from most countries while still having a reasonable timeframe (in line with follow-up times of randomised trials).^9^ Additionally, AAPCs in incidence and mortality were calculated for the time periods 1978–1987, 1988–1997, 1998–2007, and 2008 onwards. If data were not available for an entire 10-year period, yet available for at least a six-year period, AAPCs for the available time period were also calculated. AAPCs with 95% confidence intervals (CIs) were estimated by determining a single best-fitting regression line through the data over the above-mentioned time periods on a logarithmic scale.^13^

Specific information on country-/region-specific analyses are described in the Supplementary Methods.

Statistical analyses were done using SAS version 9.4, R version 4.2.1, and Joinpoint regression software (version 4.7.0.0) provided by the US National Cancer Institute (for estimation of AAPCs).

### Role of the funding source

There was no funding source for this study.

## Results

### Changes over time in incidence and mortality

In total, we included 3,420,902 female cases of invasive breast cancer and 211,804 of in situ breast cancer (Table 1).

**Fig. 1:**
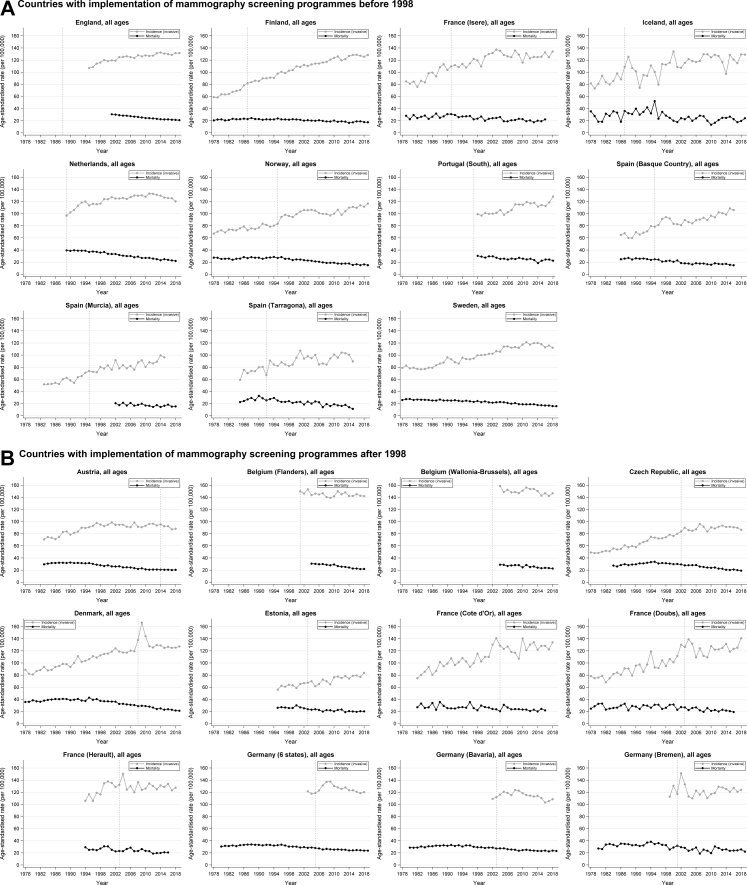

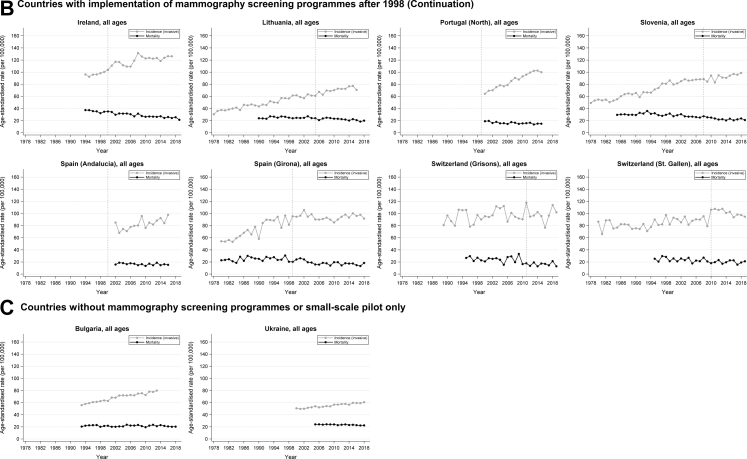
**(A–C) Changes over time in age-standardised incidence of invasive breast cancer and mortality from breast cancer by country/region. Vertical dashed lines represent years in which screening programmes were implemented.**

Over the past four decades, age-standardised incidence rates of breast cancer increased and age-standardised mortality rates decreased in the large majority of European countries (Table 2; Fig. 1). The largest increases in incidence were seen in 1978–1987 and 1988–1997. Between 2008 and 2018/19, incidence rates remained relatively stable in most countries and regions, increased in England, Finland, Norway, Portugal (North), Spain (Basque Country), Estonia, France (Doubs), Slovenia, and Ukraine, and decreased in the Netherlands, Denmark, and Germany (6 states combined and Bavaria). Decreases in mortality rates were most predominantly seen in the last two decades (1998–2007 and 2008–2018/19) (Table 2; Fig. 1).

**Table 2:** Average annual percent changes (AAPCs) in age-standardised incidence of invasive breast cancer and mortality from breast cancer for 1978–1987, 1988–1997, 1998–2007, and 2008–2018/19, by country/region.

Country/region	Measure	1978–1987	1988–1997	1998–2007	2008–2018/19
**Countries with implementation of mammography screening programmes before 1998**					
England	Incidence	–	–	0.83 (0.41–1.26)	0.33 (0.10–0.57)
	Mortality	–	–	−2.21 (−2.48 to −1.94)^a^	−1.92 (−2.14 to −1.70)
Finland	Incidence	3.59 (2.79–4.39)	2.02 (1.49–2.55)	1.28 (0.88–1.68)	0.68 (0.29–1.07)
	Mortality	1.10 (0.20–2.01)	−0.1 (−1.40 to 0.38)	−1.29 (−1.95 to −0.63)	−0.65 (−1.59 to 0.30)
France (Isere)	Incidence	2.33 (0.21–4.49)^b^	0.85 (−0.12 to 1.83)	1.00 (−0.56 to 2.58)	0.13 (−0.90 to 1.17)
	Mortality	1.12 (−2.51 to 4.88)^b^	−1.17 (−3.61 to 1.32)	−2.03 (−4.87 to 0.90)	−1.21 (−3.92 to 1.57)^c^
Iceland	Incidence	2.79 (0.61–5.01)	−1.11 (−5.17 to 3.13)	0.19 (−1.48 to 1.88)	−0.38 (−1.98 to 1.25)
	Mortality	1.29 (−6.23 to 9.41)	0.28 (−5.43 to 6.33)	0.53 (−3.65 to 4.88)	0.90 (−3.16 to 5.13)
Netherlands	Incidence	–	2.18 (0.76–3.61)^d^	0.70 (0.19–1.22)	−0.63 (−1.10 to −0.16)
	Mortality	–	−0.81 (−1.29 to −0.32)^d^	−2.69 (−3.22 to −2.15)	−2.46 (−3.07 to −1.85)
Norway	Incidence	1.14 (0.32–1.97)	2.62 (1.31–3.94)	0.70 (−0.29 to 1.69)	1.59 (1.06–2.11)
	Mortality	−0.00 (−1.27 to 1.28)	−0.21 (−1.25 to 0.84)	−2.56 (−3.23 to −1.89)	−2.11 (−2.84 to −1.38)
Portugal (North)	Incidence	–	–	3.58 (2.51–4.66)^e^	2.13 (1.09–3.18)^e^
	Mortality	–	–	−2.67 (−5.97 to 0.74)^e^	−1.07 (−3.30 to 1.22)^e^
Portugal (South)	Incidence	–	–	0.63 (0.02–1.24)	0.43 (−0.36 to 1.23)
	Mortality	–	–	−2.18 (−3.22 to −1.14)	−1.51 (−3.45 to 0.47)
Spain (Basque Country)	Incidence	–	4.42 (3.31–5.55)	−0.20 (−1.56 to 1.17)	2.02 (1.01–3.05)^f^
	Mortality	–	−1.76 (−3.01 to −0.50)	−3.18 (−4.82 to −1.51)	−1.29 (−2.98 to 0.43)^f^
Spain (Murcia)	Incidence	–	2.83 (1.16–4.54)	−0.20 (−1.69 to 1.32)	1.60 (−0.55 to 3.80)^h^
	Mortality	–	–	−2.11 (−9.80 to 6.23)^g^	−1.50 (−3.47 to 0.51)
Spain (Tarragona)	Incidence	–	1.90 (−0.10 to 3.93)	0.24 (−2.12 to 2.65)	1.03 (−1.75 to 3.88)^i^
	Mortality	–	−2.56 (−4.82 to −0.25)	−2.60 (−6.19 to 1.13)	−5.40 (−9.70 to −0.89)^i^
Sweden	Incidence	0.08 (−0.66 to 0.84)	0.72 (−0.31 to 1.76)	1.70 (1.16–2.24)	−0.06 (−0.72 to 0.60)
	Mortality	−0.69 (−1.32 to −0.06)	−1.27 (−1.85 to −0.69)	−1.39 (−2.29 to −0.48)	−2.24 (−2.69 to −1.79)
**Countries with implementation of mammography screening programmes after 1998**					
Austria	Incidence	–	2.04 (1.19–2.91)	−0.07 (−0.79 to 0.65)	−0.42 (−1.08 to 0.25)
	Mortality		−0.87 (−1.43 to −0.31)	−1.84 (−2.38 to −1.29)	−0.91 (−1.38 to −0.45)
Belgium (Flanders)	Incidence	–	–	−0.46 (−1.46 to 0.56)^j^	0.09 (−0.42 to 0.60)
	Mortality	–	–	–	−3.18 (−3.76 to −2.60)
Belgium (Wallonia and Brussels)	Incidence	–	–	–	−0.44 (−1.03 to 0.16)
	Mortality	–	–	–	−2.21 (−3.17 to −1.24)
Czech Republic	Incidence	2.27 (1.43–3.11)	3.14 (2.09–4.19)	2.38 (1.46–3.31)	0.06 (−0.64 to 0.76)
	Mortality	–	1.44 (0.44–2.45)	−1.74 (−2.33 to −1.14)	−2.68 (−3.23 to −2.14)
Denmark	Incidence	1.25 (0.26–2.25)	1.49 (0.48–2.52)	0.52 (−0.09 to 1.12)	−1.56 (−2.77 to −0.33)
	Mortality	1.43 (0.81–2.06)	0.00 (−1.04 to 1.05)	−2.54 (−3.15 to −1.93)	−3.08 (−3.64 to −2.52)
Estonia	Incidence	–	–	1.12 (−0.25 to 2.50)	1.58 (0.56–2.62)
	Mortality	–	–	−2.93 (−4.61 to −1.22)	−1.15 (−2.34 to 0.06)
France (Cote d’Or)	Incidence	2.39 (−2.26 to 7.27)^k^	−0.00 (−1.29 to 1.30)	1.74 (−0.43 to 3.95)	0.90 (−0.68 to 2.50)
	Mortality	−2.02 (−12.23 to 9.36)^k^	−0.72 (−4.22 to 2.92)	−0.20 (−3.68 to 3.40)	−1.00 (−2.96 to 0.99)^l^
France (Doubs)	Incidence	1.56 (−0.13 to 3.28)	1.75 (−0.89 to 4.45)	2.79 (0.18–5.47)	1.48 (0.32–2.65)
	Mortality	0.68 (−2.81 to 4.21)	1.26 (−1.92 to 4.54)	−2.22 (−4.93 to 0.58)	−1.13 (−3.88 to 1.70)^m^
France (Herault)	Incidence	–	–	−0.08 (−2.02 to 1.90)	−0.25 (−1.01 to 0.51)
	Mortality	–	–	−1.84 (−4.72 to 1.12)	−1.54 (−2.77 to −0.30)^n^
Germany (6 states)	Incidence	–	–	–	−1.43 (−1.77 to −1.09)
	Mortality	1.24 (0.60–1.88)^o^	−0.32 (−0.73 to 0.10)	−1.61 (−2.16 to −1.06)	−0.98 (−1.24 to −0.72)
Germany (Bavaria)	Incidence	–	–	1.45 (−0.55 to 3.50)^p^	−1.59 (−2.07 to −1.11)
	Mortality	1.59 (0.82–2.37)^o^	−0.38 (−1.13 to 0.39)	−1.42 (−1.92 to −0.92)	−0.68 (−1.15 to −0.22)
Germany (Bremen)	Incidence	–	–	−0.81 (−4.02 to 2.51)^q^	0.64 (−0.29 to 1.57)
	Mortality	3.45 (0.11–6.91)^o^	0.87 (−0.73 to 2.49)	−3.42 (−6.64 to −0.10)	−0.38 (−2.54 to 1.82)
Ireland	Incidence	–	–	1.60 (0.39–2.82)	−0.25 (−0.96 to 0.46)^r^
	Mortality	–	–	−1.90 (−3.28 to 0.51)	−2.05 (−3.05 to −1.04)
Lithuania	Incidence	3.49 (1.88–5.12)	2.76 (1.41–4.12)	1.06 (−0.05 to 2.17)	0.96 (−0.04 to 1.97)^t^
	Mortality	–	1.83 (−0.04 to 3.74)^s^	−0.91 (−2.46 to 0.67)	−2.38 (−3.28 to −1.48)
Slovenia	Incidence	1.91 (0.53–3.30)	2.39 (0.92–3.89)	0.94 (0.23–1.65)	1.22 (0.34–2.11)
	Mortality	–	−0.14 (−2.11 to 1.88)	−2.03 (−3.17 to −0.88)	−1.75 (−2.94 to −0.55)
Spain (Andalucia)	Incidence	–	–	0.04 (−5.76 to 6.20)^u^	1.38 (−1.05 to 3.88)^u^
	Mortality	–	–	0.43 (−4.29 to 5.38)^u^	0.64 (−2.55 to 3.93)^u^
Spain (Girona)	Incidence	4.55 (2.56–6.57)^v^	3.63 (0.25–7.13)	1.41 (−0.76 to 3.63)	0.74 (−0.18 to 1.68)
	Mortality	2.37 (−3.54 to 8.64)^v^	0.41 (−2.32 to 3.21)	−3.50 (−6.87 to 0.01)	−0.87 (−3.88 to 2.23)
Switzerland (St. Gallen)	Incidence	0.18 (−3.84 to 4.37)^w^	0.92 (−0.79 to 2.66)	−0.31 (−1.87 to 1.27)	0.15 (−1.53 to 1.86)
	Mortality	–	–	−2.12 (−5.46 to 1.34)	−1.31 (−4.05 to 1.51)
Switzerland (Grisons)	Incidence	–	0.14 (−4.07 to 4.54)^x^	0.86 (−1.40 to 3.18)	0.42 (−1.71 to 2.58)
	Mortality	–	–	0.24 (−4.67 to 5.40)	−3.23 (−7.59 to 1.34)
**Countries without mammography screening programmes or only pilot programmes during the study period**					
Bulgaria	Incidence	–	–	1.89 (1.20–2.57)	1.36 (−0.34 to 3.08)^y^
	Mortality	–	–	0.96 (−0.20 to 2.14)	−0.65 (−1.92 to 0.64)
Ukraine	Incidence	–	–	1.02 (0.37–1.67)^z^	1.03 (0.69–1.38)
	Mortality	–	–	–	−0.71 (−1.08 to −0.35)

If data were available for at least a 6-year period (out of the 10-year periods), AAPCs for such time periods were also calculated. If data for such time periods were not available, AAPCs were not calculated (marked as “–”).

AAPC, average annual percent change.

a For England, mortality data were only available starting from 2001 (here AAPC is shown for 2001–2007).

b For France (Isere), incidence and mortality data were available starting from 1979 only (here AAPCs are shown for 1979–1987).

c For France (Isere), mortality data were available up to 2016 only (here AAPC is shown for 2008–2016).

d For the Netherlands, incidence and mortality data were available starting from 1989 only (here AAPCs are show for 1989–1997).

e For Portugal (North), incidence and mortality data were available from 2000 to 2015 only (here AAPCs are shown for 2000–2007 and 2008–2015).

f For Spain (Basque Country), incidence and mortality data were only available up to 2016 (here AAPCs are shown for 2008–2016).

g For Spain (Murcia), mortality data were only available starting from 2002 only (here AAPC is shown for 2002–2007).

h For Spain (Murcia), incidence data were only available up to 2015 (here AAPC is shown for 2008–2015).

i For Spain (Tarragona), incidence and mortality data were available up to 2015 only (here AAPCs are shown for 2008–2015).

j For Belgium (Flanders), incidence data were available starting from 2001 only (here AAPC is shown for 2001–2007).

k For France (Cote d’Or), incidence and mortality data were available starting from 1982 only (here AAPCs are shown for 1982–1987).

l For France (Cote d’Or), mortality data were only available up to 2016 (here AAPC is shown for 2008–2016).

m For France (Doubs), mortality data were available up to 2016 only (here AAPC is shown for 2008–2016).

n For France (Herault), mortality data were available up to 2016 only (here AAPC is shown for 2008–2016).

o For Germany (all included states), mortality data were available starting from 1980 only (here AAPCs are shown for 1980–1987).

p For Germany (Bavaria), incidence data were available from 2002 only (here AAPC is shown for 2002–2007).

q For Germany (Bremen), incidence data were available from 1999 only (here AAPC is shown for 1999–2007).

r For Ireland, incidence data were available up to 2017 only (here AAPC is shown for 2008–2017).

s For Lithuania, mortality data were available from 1990 only (here AAPC is shown for 1990–1997).

t For Lithuania, incidence data were available up to 2016 only (here AAPC is shown for 2008–2016).

u For Spain (Andalucia), incidence and mortality data were only available from 2002 to 2016 (here AAPCs are shown for 2002–2007 and 2008–2016).

v For Spain (Girona), incidence and mortality data were only available starting in 1980 (here AAPCs are shown for 1980–1987).

w For Switzerland (St. Gallen), incidence data were only available starting in 1980 (here AAPC is shown for 1980–1987).

x For Switzerland (Grisons), incidence data were only available starting in 1989 (here AAPC is shown for 1989–1997).

y For Bulgaria, incidence data were available up to 2013 only (here AAPC is shown for 2008–2013).

z For Ukraine, incidence data were only available starting from 2000 (here AAPC is shown for 2000–2007).

In countries/regions with available data, age-standardised incidence rates in the 10 years before screening implementation either increased or remained stable, with no significant changes in AAPCs (Table 3). Similar incidence patterns were observed in countries with both early (pre-1998) and more recent screening implementation. Following screening implementation, incidence rates typically increased or stabilised over the next decade in many countries, with significant declines seen only in Austria and the Flemish region of Belgium. In some countries, particularly Denmark, Germany, and the Netherlands, incidence initially rose sharply before declining in subsequent years. In contrast, Bulgaria and Ukraine, where no national screening or only a small-scale pilot programmes existed, experienced a steady increase in age-standardised breast cancer incidence over time (Fig. 1).

**Table 3:** Average annual percent changes (AAPCs) in age-standardised incidence of invasive breast cancer and mortality from breast cancer for the 10-year period preceding screening implementation (“Before”) and for the 10-year period starting from the year prior to screening implementation (“After”), by country/region.

Country/region	Time period^a^	Incidence of invasive breast cancer	Mortality from breast cancer
**Countries with implementation of mammography screening programmes before 1998**			
Finland	Before (1978–1986)	3.26 (2.40–4.12)	1.25 (0.12–2.39)
	After (1986–1995)	2.04 (1.56–2.53)	−0.42 (−1.32 to 0.50)
France (Isere)	Before (1981–1990)	3.80 (2.08–5.54)	0.83 (−1.88 to 3.61)
	After (1990–1999)	1.00 (−0.04 to 2.05)	−2.57 (−4.44 to −0.67)
Iceland	Before (1978–1986)	1.91 (−0.43 to 4.31)	−0.55 (−9.48 to 9.25)
	After (1986–1995)	−0.49 (−4.22 to 3.39)	6.24 (0.61 to 12.18)
Netherlands	Before (no data)	–	–
	After (1989–1997)	2.18 (0.76–3.61)	−0.81 (−1.29 to −0.32)
Norway	Before (1985–1994)	0.81 (−0.12 to 1.76)	0.35 (−0.60 to 1.31)
	After (1994–2003)	2.86 (1.77–3.96)	−2.44 (−3.25 to −1.62)
Portugal (South)	Before (no data)	–	–
	After (1998–2005)	0.47 (−0.51 to 1.45)	−2.26 (−3.81 to −0.69)
Spain (Basque Country)	Before (1986–1994)	2.20 (0.10–4.35)	−0.56 (−1.81 to 0.71)
	After (1994–2003)	0.63 (−1.11 to 2.41)	−2.83 (−4.41 to −1.22)
Spain (Murcia)	Before (1985–1994)	2.84 (1.20–4.51)	–
	After (1994–2003)	1.84 (0.33–3.37)	–
Spain (Tarragona)	Before (1985–1991)	3.95 (0.50–7.52)	4.67 (0.50–9.05)
	After (1991–2000)	1.67 (−0.49 to 3.87)	−2.98 (−4.63 to −1.29)
**Countries with implementation of mammography screening programmes after 1998**			
Austria	Before (2004–2013)	0.22 (−0.42 to 0.96)	−2.20 (−2.82 to −1.57)
	After (2013–2018)	−1.61 (−2.85 to −0.35)	−0.58 (−1.20 to 0.03)
Belgium (Flanders)	Before (no data)	–	–
	After (2001–2009)	−0.82 (−1.43 to −0.20)	−1.67 (−2.61 to −0.73)^b^
Belgium (Wallonia and Brussels)	Before (no data)	–	–
	After (2004–2010)	−0.70 (−1.82 to 0.42)	−1.61 (−3.88 to 0.71)
Czech Republic	Before (1992–2001)	1.67 (0.91 to 2.45)	−0.85 (−1.66 to −0.03)
	After (2001–2010)	0.97 (−0.25 to 2.21)	−2.55 (−3.25 to −1.85)
Denmark	Before (1998–2007)	0.52 (−0.09 to 1.12)	−2.54 (−3.15 to −1.93)
	After (2007–2016)	−1.06 (−3.43 to 1.36)	−3.14 (−3.97 to −2.30)
Estonia	Before (1995–2002)	1.59 (−0.13 to 3.34)	0.08 (−2.15 to 2.36)
	After (2002–2011)	1.45 (−0.15 to 3.09)	−1.60 (−2.94 to −0.24)
France (Cote d’Or)	Before (1994–2003)	3.10 (0.86–5.38)	−1.68 (−4.86 to 1.60)
	After (2003–2012)	−0.59 (−2.75 to 1.62)	−1.23 (−4.17 to 1.81)
France (Doubs)	Before (1993–2002)	1.79 (−0.93 to 4.58)	−0.97 (−3.93 to 2.09)
	After (2002–2011)	−1.24 (−3.19 to 0.74)	−2.29 (−4.79 to 0.28)
France (Herault)	Before (1994–2002)	3.18 (1.33–5.05)	−0.71 (−4.18 to 2.88)
	After (2002–2011)	−0.43 (−2.08 to 1.26)	0.14 (−2.11 to 2.44)
Germany (6 states)	Before (1995–2004)	–	−1.74 (−2.30 to −1.17)
	After (2004–2013)	0.83 (−0.46 to 2.12)	−1.19 (−1.69 to −0.68)
Germany (Bavaria)	Before (1993–2002)	–	−1.58 (−2.35 to −0.81)
	After (2002–2011)	0.74 (−0.14 to 1.63)	−1.81 (−2.53 to −1.09)
Germany (Bremen)	Before (1991–2000)	–	−2.00 (−4.72 to 0.79)
	After (2000–2009)	−1.69 (−3.92 to 0.58)	−3.29 (−6.26 to −0.23)
Ireland	Before (1994–1999)	2.94 (1.09–4.82)	−2.30 (−4.60 to 0.06)
	After (1999–2008)	5.03 (3.01–7.09)	−1.84 (−3.27 to −0.40)
Lithuania	Before (1995–2004)	1.68 (0.35–3.03)	−0.34 (−1.56 to 0.90)
	After (2004–2013)	2.07 (1.25–2.90)	−0.58 (−1.90 to 0.77)
Portugal (North)	Before (no data)	–	–
	After (2000–2007)	3.58 (2.51–4.66)	−2.67 (−5.97 to 0.74)
Slovenia	Before (1998–2007)	0.94 (0.23–1.65)	−2.03 (−3.17 to −0.88)
	After (2007–2016)	1.00 (−0.11 to 2.13)	−2.66 (−3.95 to −1.36)
Spain (Andalucia)	Before (no data)	0.64 (−3.32 to 4.75)	−1.48 (−5.57 to 2.80)
	After (2002–2008)	2.20 (0.10–4.35)	−0.56 (−1.81 to 0.71)
Spain (Girona)	Before (1989–1998)	2.18 (−1.34 to 5.83)	−0.46 (−3.71 to 2.90)
	After (1998–2007)	0.21 (−1.62 to 2.06)	−3.50 (−6.87 to 0.01)
Switzerland (St. Gallen)	Before (2000–2009)	−0.56 (−2.16 to 1.07)	−0.96 (−4.52 to 2.73)
	After (2009–2018)	0.30 (−2.18 to 2.85)	−0.60 (−4.05 to 2.98)
Switzerland (Grisons)	Before (2001–2010)	−1.25 (−3.44 to 0.99)	−1.46 (−7.78 to 5.29)
	After (2010–2019)	0.11 (−3.06 to 3.39)	−0.40 (−4.93 to 4.36)

AAPC, average annual percentage change.

a If data were available for at least a 6-year period (out of the 10-year period), AAPCs for such time periods were also calculated. If data for such time periods were not available, AAPCs were not calculated (marked as “–”). No data for England and Sweden were therefore included here. In Sweden, screening was introduced in 1974; given that our study considers data available from 1978 only, AAPCs for periods right before and right after screening implementation were not available for calculation. Furthermore, Bulgaria and Ukraine were not included here because screening had not yet been implemented or only pilot programmes were available.

b For Belgium, mortality data are only available from 2004 on (here AAPC 2004–2009).

As regards breast cancer mortality, no progress was observed before introduction of screening (increases or no statistically significant changes in AAPCs) among countries with early programmes before 1998 and data available (Table 3; Fig. 1). During the first 10 years post-screening implementation, age-standardised mortality rates declined significantly in all countries (AAPCs from −2.98 [95% CI −4.63, −1.29] to −0.81 [−1.29, −0.32]) besides Finland and Iceland (where substantial decreases also occurred later) (Table 3; Fig. 1).

In Austria, the Czech Republic, Denmark, Germany, and Slovenia, where screening was implemented after 1998, significant decreases in age-standardised breast cancer mortality were already evident before screening implementation (AAPCs from −2.54 [95% CI −3.15, −1.93] to −0.85 [−1.66, −0.03]). In contrast, mortality remained largely stable in the decade preceding screening in the remaining countries (Table 3).

During the first decade of screening, significant mortality reductions were observed in Belgium (Flanders), the Czech Republic, Denmark, Estonia, Germany, Ireland, and Slovenia (AAPCs from −3.29 (95% CI −6.26, −0.23) to −1.19 (−1.69, −0.68)), while changes in AAPC in other countries, including Austria (where breast cancer mortality had decreased before screening implementation), were not statistically significant. Overall, countries with later screening rollout did not exhibit marked shifts in mortality trends between the pre- and post-screening periods.

In Bulgaria and Ukraine, where no national screening or only pilot programmes existed, mortality declines were minimal or absent (Fig. 1).

### Changes over time in stage-specific incidence

**Fig. 2:**
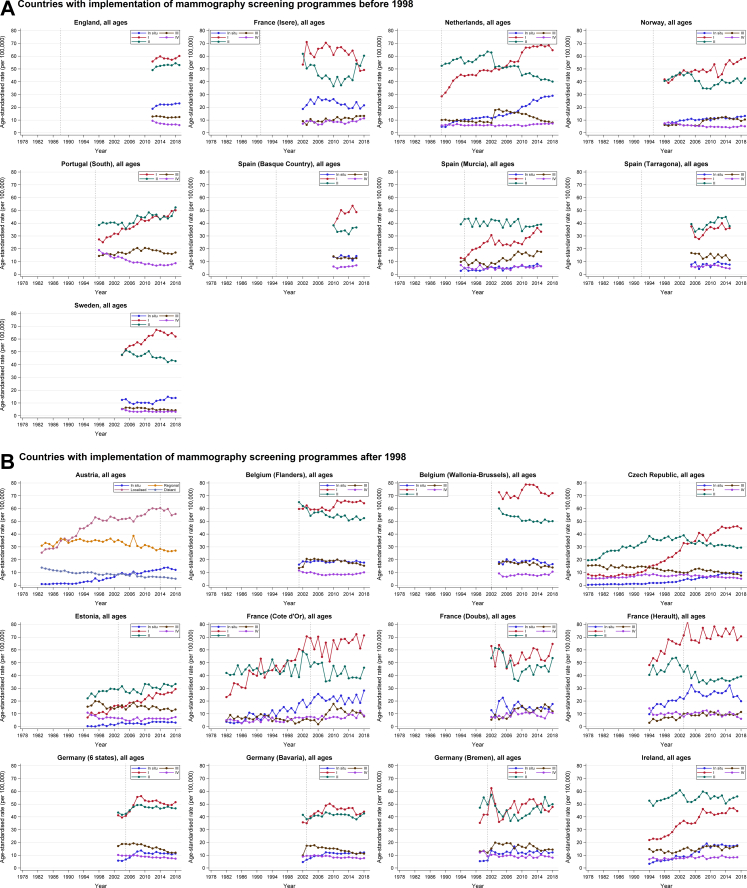

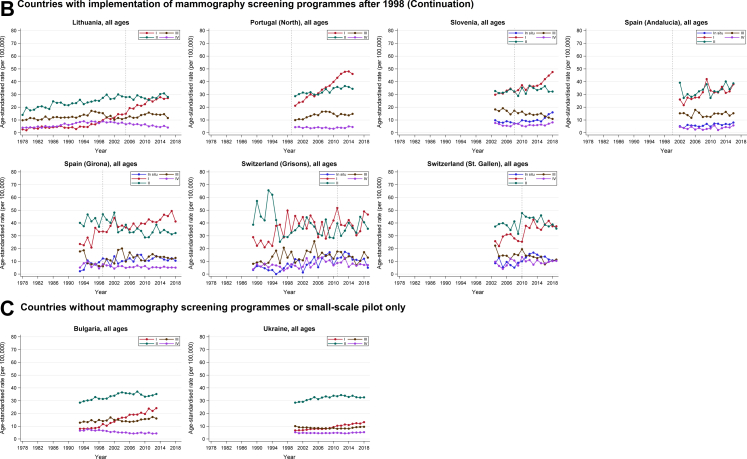
**(A–C) Changes over time in age-standardised incidence of in situ, stage I, stage II, stage III, and stage IV breast cancer, by country/region. Vertical dashed lines represent years in which screening programmes were implemented.**

Overall, the largest changes of incidence were found for in situ, stage I, and stage IV cancer (Table 4; Fig. 2). Specifically, for time periods after screening implementation, we observed substantial increases in incidence of in situ and stage I cancer for all countries with screening programmes in place before 1998 and available data, as well as for most countries whose screening programmes were implemented later (AAPCs up to +10 in the first 10 years of screening) (Table 4; Fig. 2). However, it should be noted that the increased incidence of in situ cancer reported for countries with available data may also partly reflect increased efforts to collect these data over time and should therefore be interpreted with caution. As for incidence of stage IV cancer, substantial declines were observed for several countries after introduction of screening—statistically significant AAPCs were found for Norway, Portugal (South), Belgium (Flanders), Czech Republic, Germany, and Lithuania, ranging from −6.16 (95% CI −8.28, −4.00) to −2.04 (−2.82, −1.25). Significant increases in stage IV cancer incidence were seen for France (Doubs) and Ireland only (5.28 [95% CI 1.15, 9.59] and 2.84 [1.35, 4.36], respectively).

**Table 4:** Average annual percent changes (AAPCs) in age-standardised incidence of in situ, stage I, stage II, stage III, and stage IV breast cancer for the 10-year period preceding screening implementation (“Before”) and for the 10-year period starting from the year prior to screening implementation (“After”), by country/region.

Country/region	Time period^a^	In situ	Stage I	Stage II	Stage III	Stage IV
**Countries with implementation of mammography screening programmes before 1998**						
Netherlands	Before (no data)	–	–	–	–	–
	After (1989–1997)	12.03 (7.40–16.86)	6.01 (3.43–8.65)	0.59 (−0.52 to 1.72)	−1.73 (−2.63 to −0.82)	−0.40 (−2.17 to 1.40)
Norway	Before (no data)	–	–	–	–	–
	After (1998–2003)	8.62 (1.01–16.80)	3.84 (0.98–6.78)	2.63 (0.70–4.59)	0.57 (−3.32 to 4.61)	−5.34 (−10.01 to −0.43)
Portugal (South)	Before (no data)	–	–	–	–	–
	After (1998–2005)	–	4.72 (2.86–6.62)	−0.72 (−2.26 to 0.86)	1.75 (−0.16 to 3.69)	−6.16 (−8.28 to −4.00)
Spain (Murcia)	Before (no data)	–	–	–	–	–
	After (1994–2003)	6.58 (−0.08 to 13.69)	9.61 (6.20–13.13)	0.06 (−1.68 to 1.84)	−3.13 (−7.76 to 1.72)	−3.16 (−8.98 to 3.02)
**Countries with implementation of mammography screening programmes after 1998**						
Austria	Before (2004–2013)	3.40 (1.41–5.44)	b	b	b	b
	After (2013–2018)	0.31 (−4.10 to 4.93)	b	b	b	b
Belgium (Flanders)	Before (no data)	–	–	–	–	–
	After (2001–2009)	0.61 (−1.02 to 2.26)	−0.31 (−0.91 to 0.29)	−1.94 (−3.08 to −0.79)	3.66 (−0.63 to 8.13)	−4.30 (−5.74 to −2.84)
Belgium (Wallonia and Brussels)	Before (no data)	–	–	–	–	–
	After (2004–2010)	0.73 (−3.28 to 4.92)	0.20 (−1.85 to 2.30)	−2.21 (−3.31 to −1.10)	−0.43 (−2.87 to 2.06)	1.17 (−5.12 to 7.86)
Czech Republic	Before (1992–2001)	10.60 (6.96–14.37)	9.68 (8.16–11.22)	0.74 (−0.38 to 1.87)	−3.65 (−4.36 to −2.95)	−1.58 (−3.04 to −0.10)
	After (2001–2010)	9.60 (6.30–13.00)	4.65 (2.44–6.91)	−2.04 (−3.14 to −0.92)	2.30 (0.23–4.41)	−2.32 (−3.90 to −0.72)
Estonia	Before (1995–2002)	29.45 (−1.37 to 69.91)	8.80 (2.80–15.15)	3.86 (1.79–5.97)	−2.80 (−7.40 to 2.02)	−5.72 (−10.92 to −0.21)
	After (2002–2011)	2.46 (−4.51 to 9.93)	4.50 (2.68–6.34)	0.62 (−1.87 to 3.17)	0.32 (−2.41 to 3.12)	−1.14 (−5.84 to 3.78)
France (Cote d’Or)	Before (1994–2003)	5.69 (0.02–11.68)	4.82 (3.17–6.50)	1.46 (−2.38 to 5.44)	−1.72 (−9.47 to 6.69)	5.74 (−1.53 to 13.55)
	After (2003–2012)	2.60 (−1.01 to 6.34)	−0.88 (−3.77 to 2.10)	−3.29 (−6.31 to −0.17)	18.14 (4.09 to 34.07)	−1.01 (−4.04 to 2.11)
France (Doubs)	Before (no data)	–	–	–	–	–
	After (2002–2011)	1.87 (−5.87 to 10.25)	−1.12 (−4.22 to 2.08)	−4.71 (−7.91 to −1.40)	12.10 (5.39–19.25)	5.28 (1.15–9.59)
France (Herault)	Before (1994–2002)	8.41 (3.84–13.18)	4.05 (2.06–6.08)	2.82 (0.37–5.32)	7.26 (−0.36 to 15.46)	−1.11 (−5.44 to 3.43)
	After (2002–2011)	0.81 (−2.18 to 3.90)	0.62 (−1.53 to 2.82)	−3.51 (−5.41 to −1.58)	2.88 (−0.62 to 6.50)	1.75 (−1.79 to 5.42)
Germany (6 states)	Before (no data)	–	–	–	–	–
	After (2004–2013)	8.32 (3.09–13.81)	3.32 (1.16–5.52)	1.38 (0.24–2.53)	−2.21 (−3.26 to −1.14)	−2.04 (−2.82 to −1.25)
Germany (Bavaria)	Before (no data)	–	–	–	–	–
	After (2002–2011)	9.67 (5.61–13.90)	3.68 (2.04–5.35)	0.79 (−0.06 to 1.65)	1.08 (−3.30 to 5.65)	−1.27 (−2.69 to 0.17)
Germany (Bremen)	Before (no data)	–	–	–	–	–
	After (2000–2009)	8.47 (1.29–16.17)	0.13 (−4.12 to 4.56)	−4.35 (−6.25 to −2.41)	3.39 (−0.59 to 7.53)	−3.00 (−5.63 to −0.30)
Ireland	Before (1994–1999)	12.04 (2.83–22.09)	2.94 (1.09–4.82)	1.55 (−0.70 to 3.84)	−2.42 (−7.63 to 3.09)	−1.10 (−8.34 to 6.71)
	After (1999–2008)	9.70 (6.33–13.16)	5.03 (3.01–7.09)	−0.25 (−1.75 to 1.28)	2.60 (0.37–4.88)	2.84 (1.35–4.36)
Lithuania	Before (1995–2004)	–	12.63 (9.13–16.24)	2.22 (0.92–3.54)	−4.79 (−7.60 to −1.89)	−0.79 (−2.56 to 1.02)
	After (2004–2013)	–	6.86 (4.86–8.91)	−0.55 (−1.59 to 0.51)	3.77 (2.07–5.51)	−4.41 (−6.40 to −2.39)
Portugal (North)	Before (no data)	–	–	–	–	–
	After (2000–2007)	–	6.30 (4.50–8.13)	0.57 (−0.74 to 1.88)	7.26 (5.33–9.23)	−2.07 (−5.61 to 1.59)
Slovenia	Before (no data)	–	–	–	–	–
	After (2007–2016)	3.75 (1.01–6.56)	2.17 (0.92–3.42)	1.17 (−0.68 to 3.06)	−1.68 (−3.47 to 0.14)	0.06 (−3.09 to 3.30)
Spain (Andalucia)	Before (no data)	–	–	–	–	–
	After (2002–2008)	1.99 (−6.13 to 10.82)	3.98 (−0.21 to 8.35)	−0.44 (−6.77 to 6.32)	−1.13 (−8.51 to 6.84)	−7.64 (−18.14 to 4.21)
Spain (Girona)	Before (1993–1998)	29.89 (−2.43 to 72.92)	8.31 (−4.21 to 22.48)	−0.15 (−6.58 to 6.73)	−20.04 (−31.09 to −7.21)	−4.21 (−23.84 to 20.49)
	After (1998–2007)	0.68 (−5.59 to 7.37)	1.41 (−0.76 to 3.63)	−2.97 (−5.93 to 0.08)	9.91 (0.94 to 19.68)	−1.36 (−6.29 to 3.82)
Switzerland (St. Gallen)	Before (2003–2009)	−5.01 (−19.53 to 12.14)	2.04 (−4.20 to 8.68)	−1.90 (−6.15 to 2.53)	−4.15 (−12.32 to 4.78)	3.50 (−14.60 to 25.43)
	After (2009–2018)	1.46 (−3.94 to 7.17)	4.12 (0.54–7.83)	−0.34 (−3.62 to 3.05)	−6.77 (−10.60 to −2.77)	0.15 (−5.23 to 5.84)
Switzerland (Grisons)	Before (2001–2010)	11.40 (−7.01 to 33.47)	−1.74 (−5.95 to 2.67)	−3.16 (−6.54 to 0.34)	0.45 (−6.35 to 7.74)	6.63 (−3.00 to 17.20)
	After (2010–2019)	−3.60 (−12.28 to 5.94)	−0.54 (−4.87 to 3.99)	2.43 (−0.98 to 5.96)	−2.25 (−9.11 to 5.12)	−2.02 (−7.41 to 3.69)

No data for England, France (Isere), Spain (Basque Country), Spain (Tarragona), and Sweden, as well as Finland, Iceland, Denmark (for these 3, we only included data publicly available from NORDCAN, which do not include stage information). Furthermore, Bulgaria and Ukraine were not included here because screening had not yet been implemented or only pilot programmes were available.

a If data for such time periods were not available, AAPCs were not calculated (“marked as “–”).

b Austria: Before screening implementation—Localised: 1.95 (1.16 to 2.73); Regional: −1.89 (−3.81 to 0.07); Distant: −3.71 (−6.40 to −0.94). After screening implementation—Localised: −1.71 (−3.44 to 0.06); Regional −0.79 (−2.23 to 0.68); Distant: −4.76 (−6.44 to −3.05).

In Bulgaria and Ukraine (countries with no screening programmes or pilot programmes only), incidence of stage I cancer also increased; incidence of stage IV cancer decreased over time in Bulgaria and remained relatively stable in Ukraine (Fig. 2).

### Changes over time in total and stage-specific incidence and mortality by age at diagnosis

Distinct patterns in incidence trends were observed between the various age groups, particularly in stage-specific analysis (Supplementary Tables S3–S8; Supplementary Figs. S1 and S2). Incidence of in situ and stage I cancer increased across all age groups in most countries, but were overall most pronounced among the group targeted by screening (mostly 50–69 years) and, for several countries, also among older age groups or already before screening was introduced (Supplementary Tables S4 and S5; Supplementary Fig. S2). Stage II cancer incidence decreased substantially in the years after screening implementation among the screening age group (mostly 50–69 years) in most countries, yet with heterogeneity across countries (Supplementary Table S6; Supplementary Fig. S2). Larger decreases were overall seen in stage III and IV cancer incidence, particularly in the age groups 50+ years (Supplementary Tables S7 and S8; Supplementary Fig. S2). Breast cancer mortality rates declined considerably especially among 0–49- and 50–69-year-olds, whereas no significant changes or even increasing rates were seen for older age groups (80+ years) in most countries (Supplementary Table S9; Supplementary Fig. S1).

## Discussion

In this international population-based study, we described changes in incidence of and mortality from breast cancer between 1978 and 2019 in light of mammography screening implementation in 21 European countries. We observed overall substantial increases in breast cancer incidence over the investigation period, which was largely driven by increases in detection of in situ and stage I cancers. Yet, we also observed considerable declines in incidence of stage IV breast cancer, particularly after introduction of mammography screening programmes. Breast cancer mortality decreased across most countries and mostly starting in the late 1990s. Despite these overall trends, we still observed large intercountry differences pointing to various progress in breast cancer control and screening effects.

Several randomised controlled trials, carried out more than 30 years ago, estimated that females invited to mammography screening had a 20% reduced risk of dying from breast cancer as compared to those not invited.^9^ More recent observational studies have reported similar estimates of mortality reduction—for example, a recent review and meta-analysis of 43 cohort studies (39 in Europe, 2 in New Zealand, 1 in the US, and 1 in Canada) showed a 22% reduction in breast cancer mortality with invitation to screening and a 33% reduction with attendance to screening.^14^ The population-level impact of mammography screening programmes has also been studied on the national and regional levels in several European countries, with evidence pointing to the contribution of screening to reductions in breast cancer mortality over the past two decades.^12^^,^^15^

Reductions in mortality are achieved due to early detection of breast cancers that would have otherwise been diagnosed at a later stage and caused death.^16^ However, alongside early detection, there is overdiagnosis (i.e., detection of cancers that would not have been diagnosed or caused death in a person's lifetime). European studies that accounted for both lead time and contemporaneous trends in incidence estimated overdiagnosis to make up 1–25% of detected cases.^17^^,^^18^

In this study, we observed marked increases in the incidence of in situ and stage I breast cancer among screening age groups (mostly 50–69 years) following the implementation of mammography screening programmes. In some countries, such as Austria and the Czech Republic, these increases began earlier, likely reflecting widespread opportunistic screening prior to organised programme rollout.^19^^,^^20^ The incidence trends among non-screening-eligible women aged 0–49 may be partly explained by increased access to care and use of mammography (as opportunist screening or for diagnostic purposes). Survey data from 2013–2015 indicate that over 30% of women under 50 had undergone a mammogram within the preceding two years in several countries, including Austria, Bulgaria (despite no organised programme was available), France, Portugal, and Sweden.^10^

In countries like Denmark, Germany, and the Netherlands, the sharp rise in incidence after introduction of mammography screening likely reflects the detection of prevalent, yet asymptomatic (mostly early-stage) cases, a well-documented and expected pattern after the introduction of population-based screening programs.^14^^,^^21^

Besides increases in incidence of early stage cancer, we observed decreases in incidence of advanced-stage cancer, particularly of stage IV and among 50+ age groups, in the years after screening implementation. These findings suggest that detection of early-stage cancer through screening has prevented a large proportion of cases from being diagnosed at a later stage when prognosis is overall less favourable.^8^^,^^22^ Thus, our findings further point to the contribution of screening to the decreasing mortality rates observed in the past 10–20 years.

Despite these overall patterns, we still observed large intercountry variations in changes in incidence after screening implementation, which might in part reflect differing speeds of screening roll out, uptake, quality control measures, and screening effects.^10^ For instance, the lack of an increase in (early-stage) breast cancer incidence right after introduction of organised screening in the Flemish region (Belgium) may be, to a certain extent, attributed to the gradual screening roll out.

The data also suggests that mammography screening has led to overdiagnosis, as the increases in incidence of in situ and stage I cancers were much larger than the decreases in incidence of advanced-stage cancers, and overall, no marked changes in incidence were observed among the post-screening age groups. Development and implementation of personalised, risk-based, screening offers on the one hand, and improvements in our understanding of the biological behaviours of breast cancers on the other hand have, however, the potential to considerably reduce overdiagnosis and overtreatment and revolutionise breast cancer screening and outcomes in the future.^23^^,^^24^

Alongside screening, high (and partly increasing) prevalence of reproductive, hormonal, and behavioural risk factors, namely fewer children, advanced age at first birth, menopausal hormone-replacement therapy, oral contraceptives, alcohol consumption, excess body weight, and physical inactivity might help explain the high (and increasing) overall incidence rates, particularly in the 1980s and 1990s.^2^, ^3^, ^4^, ^5^

Regarding mortality, substantial improvements in breast cancer treatment and prognosis in the past two decades, via universal health care coverage and availability and quality of care in public cancer centres are likely to have made an even larger contribution to the observed declines than screening. This is supported by the lack of a substantial shift in mortality between pre- and post-screening periods in countries with more recent screening implementation, and is concordant with a recently published modelling study from the USA, which estimated that from 1975 to 2020, screening accounted for 25% of breast cancer deaths averted, while treatment advances accounted for 75%.^25^ It should also be noted that screening may only start impacting mortality about five years after its implementation as reported in RCTs.^26^

Conversely, in Bulgaria and Ukraine (both countries with no mammography screening programmes or only pilot programmes), limited health care resources and absence of operational national cancer plans to deliver high quality care and treatment might provide major reasons for the lack of or only minor progress against mortality.^27^^,^^28^

It is also important to bear in mind that mammography screening has evolved over the years^7^ and is continuously evolving. Specifically, the use of artificial intelligence to support, for example, screen reading might improve detection and also allow application in countries and settings where the need for numerous qualified human resources has made screening implementation difficult so far.^29^ However, such improvements in detection should also be assessed in relation to potential additional harms, namely overdiagnosis and overtreatment. Furthermore, adaptations to the guidelines and screening programmes are expected to take place in the near future, following the new 2022 recommendations of the Council of the European Union, which suggest lowering the starting age of screening to 45 and extending the age to stop screening to 74.^30^ Nevertheless, countries should assess the local context, resources, and cost-effectiveness, particularly in settings where organised screening is not yet available. Continuous monitoring and assessment of mammography screening effects ought to take place in light of these developments as well as improvements in treatment.

This study is, to our knowledge, the most comprehensive analysis of breast cancer incidence and mortality changes in the last three to four decades in Europe, providing detailed data by stage and age at diagnosis and examining the potential impact of mammography screening on the observed trends. Nonetheless, several limitations should be acknowledged. First, the availability of data varied across countries; however, including data from earlier years where possible allowed us to assess cancer burden trends around the time of screening implementation, including in countries that began before the 1990s or even the 1980s. Second, while completeness of staging data differed across registries and countries, missing or unknown stage was under 20% in all countries except Germany, Portugal (North), and Spain (Tarragona). Also, missing or unknown staging information (whose proportion varied over time across countries) was imputed to minimise biases in the interpretation of trends in stage-specific incidence. Nevertheless, stage-specific incidence trends should be interpreted with caution, especially in countries with a high proportion of unknown stage. Third, a likely stage migration (from stage II to III) might have occurred over time, particularly around 2003, due to reclassification of tumours with more than three positive lymph nodes as stage III instead of stage II. This was noticeable for the Netherlands, Belgium (Flanders), and Germany among 0–49-year-olds. Fourth, in situ case data were unavailable for seven countries, and the increased incidence of in situ cancer reported for countries with available data may also partly reflect increased efforts to collect these data. Fifth, while our study offers valuable real-world insights into breast cancer trends across screening contexts, it does not allow quantification of screening's specific contribution to the observed trends or direct comparison with randomised trial estimates. Lastly, we were unable to collect data on race and ethnicity. Therefore, the representativeness for minority groups was affected and we were unable to take sociocultural constructs and potential marginalisation into account.

In summary, this study suggests that mammography screening programmes have contributed to the patterns and trends in breast cancer burden in European countries. Specifically, the data point to the role of screening in increases in detection of in situ and stage I cancer alongside decreases in detection of advanced stage (particularly stage IV) cancer and thereby its contribution to the observed decreases in breast cancer mortality rates over the past 20 years across European countries. Despite these overall trends and patterns, we still found substantial heterogeneity across countries that suggest varying effects of mammography screening programmes. This study provides important evidence for further research, implementation, and roll out of breast cancer screening in European countries.

## Contributors

H.B. and R.Car. conceived and designed the study. R.Car. did the literature search. M.Ha., P.I., J.F., N.V.D., Z.V., T.A., O.M., O.N., K.I., M.Mä., S.D.Y., A.-S.W., B.T., F.P., P.M.W., I.V., L.S., L.d.M., S.S., T.B.J., S.H., R.Cal., M.J.B., F.A.d.C., A.M., A.L., T.Ž., S.T., M.R.-B., M.-J.S., A.L.M.M., R.M.-G., A.S., A.S.G., M.-D.C.L., J.G., F.S., K.S., J.S., M.-Å.P., M.B., M.Mo., R.v.M., O.S., and A.R. contributed to preparation of the national and regional datasets. R.Car. carried out the analysis. R.Car. and I.O. drafted the manuscript and carried out co-authors review and editing. All authors contributed to the interpretation of the results and critically revised the manuscript. R.Car., I.O. and H.B. had full access to the raw data, verified the data, and take responsibility for the integrity and accuracy of the analyses. All authors had full access to all the findings reported in the study and accept responsibility to submit the paper for publication.

## Data sharing statement

The authors signed confidentiality statements for access to and analysis of the raw data, which makes it impossible to make them publicly available. The data used for this study are, however, available at the registries and can be applied for (information for the various registries can be found in Supplementary Table S1).

## Declaration of interests

J.F. and N.V.D. are employed by the Belgian Cancer Registry, which is financed by regional and federal authorities for collecting data regarding new cancer diagnoses and cancer screening in Belgium, and for disseminating associated epidemiological parameters. R.Car. is employed by The Lancet Regional Health—Europe at the time of submission of this article for publication but not during the analyses and manuscript preparation. F.A.d.C. reports consulting fees for WHO Regional Office for Europe, and support by Sringer for attending EUPHA 2024. R.v.M. reports grants or contracts from BMS, MSD, Astra Zeneca, and All Can; payment or honoraria from Amgen, Novartis, and Roche; support for attending meetings and/or travel from Takeda and Pharmamar; patents planned, issued, or pending from Pharmamar; and participation on a data safety monitoring board or advisory board for Amgen Astra Zeneca, Bayer, BMS, Elli Lilly, GSK, Merck Serono, MSD, Novartis, Pharmamar, Pfizer, Sanofi, Roche, Vifor, and Immunophotonics. All other authors have no competing interests to disclose. SS reports consulting fees for the Pfizer Boost grant application, and being part of the Advisory Board of the Netherlands Epidemiology Society and the Advisory Board of Evidencio.
