# Supplementary material for: Breast cancer incidence, by stage at diagnosis, and mortality in 21 European countries in the era of mammography screening: an international population-based study
Source: Lancet Reg Health Eur. 2025 Dec 29;62:101574. doi: 10.1016/j.lanepe.2025.101574 (PMC12803884; doi:10.1016/j.lanepe.2025.101574)
Supplement: Supplementary Information [file mmc1.docx]

**SUPPLEMENTARY APPENDIX**

**Table of contents**

**Page**

Table of contents 1

Supplementary methods 2-3

Supplementary Table 1. Data sources and data quality indicators, by country/ region. 4-6

Supplementary Table 2. Characteristics and parameters of breast cancer screening programmes in the countries included in the study. 7-10

Supplementary Table 3. Average annual percent changes (AAPCs) in age-standardised incidence of invasive breast cancer for the 10-year period preceding screening implementation (“Before”) and for the 10-year period starting from the year prior to screening implementation (“After”), by age at diagnosis. 11

Supplementary Table 4. Average annual percent changes (AAPCs) in age-standardised incidence of in situ breast cancer for the 10-year period preceding screening implementation (“Before”) and for the 10-year period starting from the year prior to screening implementation (“After”), by age. 12

Supplementary Table 5. Average annual percent changes (AAPCs) in age-standardised incidence of stage I breast cancer for the 10-year period preceding screening implementation (“Before”) and for the 10-year period starting from the year prior to screening implementation (“After”), by age. 13

Supplementary Table 6. Average annual percent changes (AAPCs) in age-standardised incidence of stage II breast cancer for the 10-year period preceding screening implementation (“Before”) and for the 10-year period starting from the year prior to screening implementation (“After”), by age. 14

Supplementary Table 7. Average annual percent changes (AAPCs) in age-standardised incidence of stage III breast cancer for the 10-year period preceding screening implementation (“Before”) and for the 10-year period starting from the year prior to screening implementation (“After”), by age. 15

Supplementary Table 8. Average annual percent changes (AAPCs) in age-standardised incidence of stage IV breast cancer for the 10-year period preceding screening implementation (“Before”) and for the 10-year period starting from the year prior to screening implementation (“After”), by age. 16

Supplementary Table 9. Average annual percent changes (AAPCs) in age-standardised breast cancer mortality for the 10-year period preceding screening implementation (“Before”) and for the 10-year period starting from the year prior to screening implementation (“After”), by age. 17

Supplementary Figure 1. Changes over time in age-standardised incidence of invasive breast cancer and mortality from breast cancer by country and age. 18-23

Supplementary Figure 2. Changes over time in age-standardised incidence of in situ, stage I, stage II, stage III, and stage IV breast cancer by country and age. 24-28

Supplementary reference list 29

**Supplementary Methods**

Inclusion of cases based on IARC/IACR reporting rules

The first invasive breast tumour of each morphological type was considered for each patient, based on the rules for reporting multiple primary cancers set out by the International Agency for Research on Cancer and International Association of Cancer Registries (IARC/IACR).^(Supplementary ref.1)^ The first in situ breast tumour of each morphological type was also counted for each patient unless it was preceded by or synchronous with an invasive tumour of the same morphological type. In situ cases that occurred before an invasive case of the same morphological type were also counted, except for Germany.

Country-specific analyses

For some countries, the data available and/or analyses differed slightly from the descriptions above, notably:

- Austria: the categories used for stage were “localized”, “regional” (metastases in lymph nodes), and “distant” (metastases in other parts of the body).
- Belgium: the regions Wallonia and Brussels were analysed together because screening was implemented in the same year in these two regions and participation (both in the context of the organised programme as well as in the opportunistic setting) was rather similar. Imputation of missing stage was done on a dataset containing all cases from all regions, and the region was also included in the imputation model.
- England: information on age at diagnosis was only available by 12 age groups (0-39, 40-44, 45-49, …, 85-89, 90+ years) and detailed topography information was not available (all cases coded as C050). Therefore, less detailed data were used for imputation of missing stage and all cases diagnosed up to age 39 were given equal weight for analyses of age-standardised incidence rates. Also, in England, cases are reported for all tumours with different morphology codes at the third digit level and not based on IARC/IACR rules.
- Estonia: stage-specific incidence analyses by age at diagnosis were not conducted because of low population numbers.
- Germany: we only included data from eight states (out of 16): Schleswig-Holstein, Hamburg, Lower Saxony, North Rhine Westphalia, Rhineland Palatinate, Saarland, Bavaria, and Bremen. For the remaining states, data were not complete for the years 2003-2019. Data from Bavaria and Bremen were analysed separately because screening was introduced earlier in these two states (in 2003 and 2001, respectively). In situ cases that occurred before an invasive case of the same morphological type were also counted. For analyses of mortality, data from all areas of North Rhine Westphalia were included; for analyses of incidence, only data from the administrative district Muenster were considered. Imputation of missing stage was done on a dataset containing all cases from the eight included states (state was included in the model), and cases diagnosed on the basis of death certificate only were excluded from analyses by stage.
- Iceland: given the low population numbers, data were only shown for all ages combined.
- Norway: data on topography were not available and could not be used in the imputation model for analyses of stage.
- Ukraine: cases diagnosed in the regions Crimea, Donetsk, and Luhansk were excluded. Coverage in these areas was <80% in recent years due to political instability.

Statistical analyses were done using SAS version 9.4, R version 4.2.1 (MICE package for imputation of staging information)^(Supplementary ref.2)^ and Joinpoint regression software (version 4.7.0.0) provided by the US National Cancer Institute (for estimation of AAPCs).^(Supplementary ref.3)^

**Supplementary Table 1.** Data sources and data quality indicators, by country/ region.

| **Country/ region** | **Data source for patient- and tumour-level data** | **Data source for population and mortality data** | **% Microscopically verified cases** ^a^ | **% DCO during study period** ^a^ | **Level of completeness (%)** ^a^ | **National/ regional coverage (%)** ^a, b^ |
| --- | --- | --- | --- | --- | --- | --- |
| **Austria** | Austrian National Cancer Registry  <https://www.statistik.at> | Statistics Austria  <https://www.statistik.at> | 75·1-93·1 | 5·2-22·5 | 94·2-100 | 100 |
| **Belgium** | Belgian Cancer Registry  <https://kankerregister.org> | Statbel, the Belgian statistical office  <https://statbel.fgov.be> | 96·7-98·5 | 0 | >95 | 100 |
| **Bulgaria ^c^** | Bulgarian National Cancer Registry  <http://www.sbaloncology.bg> | Republic of Bulgaria National Statistical Institute  <https://www.nsi.bg> | 65·2-86·1 | 4·8-13·7 | 91·1-96·0 | 100 |
| **Czech Republic** | Czech National Cancer Registry  <https://www.svod.cz> | Czech National Cancer Registry  <https://www.svod.cz> (mortality data) Czech Statistical Office  <https://www.czso.cz> (population data) | 70-90 | 0-6 | 95 | 100 |
| **England** | National Cancer Registration and Analysis Service, Public Health England <https://www.gov.uk/government/organisations/public-health-england> | Office for National Statistics, UK  <https://www.ons.gov.uk> | 80-85 (between 2002 and 2019) | 0·5-5 (between 2002 and 2019) | >99 | 100 |
| **Estonia** | Estonian Cancer Registry  <https://www.tai.ee/et/statistika-ja-registrid/vahiregister> | Statistics Estonia  <https://www.stat.ee> | 84·6-90·7 | 0·58-2·65 | ~95 | 100 |
| **France** |  |  |  |  |  |  |
| Cote d’Or | Registry of Breast and Gynaecological Cancers of Cote d'Or | National Institute of Statistics and Economic Studies, France  <https://www.insee.fr> | 90-95 | 0 | >95 | National  0·83;  Regional  100 |
| Doubs | Doubs Tumour Registry | National Institute of Statistics and Economic Studies, France  <https://www.insee.fr> | 90-95 | 0 | >95 | National  0·84; Regional  100 |
| Herault | Herault Tumour Registry | National Institute of Statistics and Economic Studies, France  <https://www.insee.fr> | 94-95 | 0 | >95 | National 1·7; Regional 100 |
| Isere | Cancer registry of Isere | National Institute of Statistics and Economic Studies, France  <https://www.insee.fr> | 95-98 | 0 | >95 | National 1·96; Regional 100 |

**Supplementary Table 1.** Continued.

| **Country/ region** | **Data source for patient- and tumour-level data** | **Data source for population and mortality data** | **% Microscopically verified cases** ^a^ | **% DCO during study period** ^a^ | **Level of completeness (%)** ^a^ | **National/ regional coverage (%)** ^a, b^ |
| --- | --- | --- | --- | --- | --- | --- |
| **Germany ^d^** | German Centre for Cancer Registry Data (ZfKD)  <https://www.krebsdaten.de> | Federal Statistical Office, Germany  <https://www.destatis.de> |  |  |  |  |
| Schleswig-Holstein |  |  | 79·7-87·9 | 2·4-16·1 | >95 | Regional 100;  National 25 |
| Hamburg |  |  | 80·0-86·0 | 2·5-11·5 | 90 - >95 |  |
| Lower Saxony |  |  | 61·9-85·9 | 6·6-20·2 | 93 - >95 |  |
| North Rhine Westphalia (administrat-ive district Muester) |  |  | 81·4-87·0 | 5·0-11·2 | 91 - >95 |  |
| Rhineland Palatinate |  |  | 48·6-69·9 | 1·5-4·8 | 80 - >95 |  |
| Saarland |  |  | 74·0-91·2 | 4·4-8·6 | 94 - >95 |  |
| Bavaria |  |  | 75·8-90·6 | 5·2-21·4 | 86 - >95 | Regional 100;  National 16 |
| Bremen |  |  | 84·5-90·2 | 3·4-13·4 | ≥95 | Regional 100;  National 0·8 |
| **Ireland** | National Cancer Registry Ireland (NCRI) https://www.ncri.ie | Central Statistics Office (CSO)  <https://www.cso.ie> | 83-92 | 0·5-3·7 | ≥95 | 100 |
| **Lithuania** | Lithuanian Cancer Registry  <https://www.nvi.lt/cancer-registry/> | Official Statistics Portal, Lithuania  <https://osp.stat.gov.lt> | 52·6-92·9 | 3·17-7·42 | ~95 | 100 |
| **Netherlands** | Netherlands Cancer Registry, Netherlands Comprehensive Cancer Organisation (IKNL)  <https://www.iknl.nl/en/ncr> | Statistics Netherlands  <https://www.cbs.nl> | 95·4-96·7 | - | >95 | 100 |
| **Norway** | Cancer Registry of Norway  <https://www.kreftregisteret.no> | Statistics Norway  <https://www.ssb.no> | 93·0 | 1·4 | 98·6 | 100 |
| **Portugal** |  |  |  |  |  |  |
| North region | North Region Cancer Registry of Portugal (RORENO)  <https://ipoporto.pt/roreno/> | Statistics Portugal  <https://www.ine.pt> | 93·0 | - | 83-96 | Regional  100 |
| South region | South Region Cancer Registry of Portugal (ROR-Sul) | Statistics Portugal  <https://www.ine.pt> | 95·1 | 0·14 | >95 | Regional 100;  National: 46 |
| **Slovenia** | Slovenian Cancer Registry  <https://www.onko-i.si/eng/crs>; <http://www.slora.si/en/> | Statistical Office of the Republic of Slovenia  <https://www.stat.si> | Between 82·4 (1978) and 94·0 (2017) | Between 0·19 (2012) and 5·1 (1991) | ~95 | 100 |

**Supplementary Table 1.** Continued.

| **Country/ region** | **Data source for patient- and tumour-level data** | **Data source for population and mortality data** | **% Microscopically verified cases** ^a^ | **% DCO during study period** ^a^ | **Level of completeness (%)** ^a^ | **National/ regional coverage (%)** ^a, b^ |
| --- | --- | --- | --- | --- | --- | --- |
| **Spain** |  |  |  |  |  |  |
| Andalucia | Granada Cancer Registry <https://www.registrocancergranada.es/> | National Statistics Institute, Spain  <https://www.ine.es> | 81·5-90·0 | 0·2-5·4 | >98 | 100 |
| Basque country | Basque Cancer Registry <https://www.euskadi.eus/informacion/registros-de-cancer/web01-a3regepi/es/> | National Statistics Institute, Spain  <https://www.ine.es> | From 77·5 (1987) to 91·1 (2015) | From 13·7 (1986)  to 0·36 (2014) | From 86·1 (1986) to 98·4 (2014) | Regional  100 |
| Girona | Girona Cancer Registry <https://ico.gencat.cat/ca/professionals/serveis_i_programes/registre_del_cancer/> | National Statistics Institute, Spain  <https://www.ine.es> | 85·0-91·7 | 0·2-5·5 | 90·5-95·8 ^e^ | National  ~1·5; Regional 100 |
| Murcia | Murcia Cancer Registry  <http://www.murciasalud.es> | National Statistics Institute, Spain  <https://www.ine.es> | 89·7 | 2·95 | 97 ^f^ | National  3·2;  Regional 100 |
| Tarragona | Tarragona Cancer Registry  <https://epicancer.cat> | Statistical Institute of Catalonia (Idescat)  <https://www.idescat.es> | 96·8 | 1·0 | ~96 | National  ~1·6;  Regional 100 |
| **Switzerland** |  |  |  |  |  |  |
| Eastern Switzerland | Cancer Registry of Eastern Switzerland, | Swiss Federal Statistical Office  <https://www.bfs.admin.ch> | 85·1-97·1 | 0·1-14·4 | >95 | National  10·0-10·4 |
| Grisons-Glarus | Cancer Registry of Grisons-Glarus  <https://www.ksgr.ch/krebsregister> | Swiss Federal Statistical Office  <https://www.bfs.admin.ch> | 91·5-95·8 | 0·0-2·6 | >95 | National 2·8-3·2 |
| **Ukraine** | National Cancer Registry of Ukraine <http://www.ncru.inf.ua> | State Statistics Service of Ukraine <http://db.ukrcensus.gov.ua/PXWEB2007/popul_eng.htm> | 73·6-82·9 | 0·4-1·2 | 94·6-95·5 | National 85;  Among included regions 100 |

DCO, death certificate only.

^a^ The lowest and highest annual percentages during the period of investigation are provided for all cancers apart from C44.

^b^ Coverage refers to the proportion of the population in the various countries/ regions that are covered by the registries.

^c^ Bulgaria: data refer to the time period 2000-2013.

^d^ Germany: % of microscopically verified cases, DCO, and level of completeness for 2003-2019; coverage refers to the calendar year 2019.

^e^ The level of completeness for Girona refers to the time period 2003-2017.

^f^ The level of completeness for Murcia refers to the time period 2000-2018.

**Supplementary Table 2.** Characteristics and parameters of breast cancer screening programmes in the countries included in the study.

| **Country/ region** | **Test(s)** | **Year of programme initiation** | **Target ages (years)** | **Screening interval (years)** | **Invitation coverage [invitation / eligible] in organised screening (year of data collection)** | **Participation rates [participation / invited] in organised screening (year of data collection)** | **Proportion of the population aged 50-69 years who have done a mammography within the preceding 2 years ^a^** |
| --- | --- | --- | --- | --- | --- | --- | --- |
| **Countries with implementation of mammography screening programmes before 1998** | | | | | | | |
| **England ^b^** | Screen-film mammography and digital mammography (by 2016, 100% digital mammography) | 1988 | 50-64 (1988-2005); 50-70 (2005 on) | 3 | Fully implemented in 1993 | 70·5-73·6% over 2008-2018 | 78·7% (2013-15) |
| **Finland** | Screen-film mammography and digital mammography (by 2016, 100% digital mammography) | 1987 | 50-59 (1987-2006); 50-69 (2007 on) | 2 | 91·6% (2012); 97·5-100% (2014-2019) | 83·0% (2012); 82·7% (2015); 82·6% (2016); 81·4% (2018); 81·6% (2019) | 86·5% (2013-15); 92·3% (2018-20) |
| **France (Isere)** | Screen-film mammography and digital mammography | 1991 | 50-69 (1991-2001); 50-74 (2002 on) | 2 | Above 95% over the period 2002-2018 (age group 50-74) | Ranging from 36·8% to 57·0% between 2005-2006 and 2018-2019 | Nationwide data: 87·0% (2013-15); 70·0% (2018-20) |
| **Iceland** | Screen-film mammography and digital mammography (timing of implementation and share of digital mammography are unknown) | 1987 | 40-69 | 2 | Fully implemented in 1989 | 60·0% (1998-2010) | 65·6% (2013-15); 71·4% (2018-20) |
| **Netherlands** | Screen-film mammography and digital mammography gradually implemented since 2003 (fully implemented in 2010) | 1989 | 50-69 (1989-1997); 50-74 (1998 on) | 2 | Fully implemented in 1997; Age groups 50-74: 101·4% (2013); 98·4% (2014); 101·3% (2015); 100·2% (2016); 100·4% (2017); 94·4 (2018); 97·1% (2019); 55·1 (2020) | 80·1% and 77·1% (50-69- and 70-74-year-olds in 2013); 76·9% (2017 and 2018); 76·0% (2019); 71·2% (2020) | 80·4% (2013-15); 77·7% (2018-20) |
| **Norway** | Screen-film mammography and digital mammography since 2000 | 1995 | 50-69 | 2 | Fully implemented in 2005 | 74·7% (2007-2014) | 75·6% (2013-15); 76·4% (2018-20) |
| **Portugal (South)** | Screen-film mammography and digital mammography (by 2016, 100% digital mammography) | 1997 (Alentejo region); 2001 (Lisbon and Tagus Valley region); 2005 (Algarve region) | 45-74 (50-69 in the Algarve region) | 2 | 55·4% (2013) | Year 2013, 50-69-year-olds: 58·5% (Alentejo); 53·7% (Lisbon) | Nationwide data: 84·2% (2013-15); 81·1% (2018-20) |

**Supplementary Table 2.** Continued.

| **Country/ region** | **Test(s)** | **Year of programme initiation** | **Target ages (years)** | **Screening interval (years)** | **Invitation coverage [invitation / eligible] in organised screening (year of data collection)** | **Participation rates [participation / invited] in organised screening (year of data collection)** | **Proportion of the population aged 50-69 years who have done a mammography within the preceding 2 years ^a^** |
| --- | --- | --- | --- | --- | --- | --- | --- |
| **Spain (Basque Country)** | Screen-film mammography and digital mammography | 1995 | 50-69 | 2 | Fully implemented in 1997 | 79% (2015-2016) | Nationwide data: 80·3% (2013-15); 74·1% (2018-20) |
| **Spain (Murcia)** | Screen-film mammography and digital mammography | 1995 | 50-69 | 2 | Fully implemented in 2002 | Ranging from 67·4% to 72·3% over the period 1995-1996 and 2014-2015 | Nationwide data: 80·3% (2013-15); 74·1% (2018-20) |
| **Spain (Tarragona)** | Screen-film mammography and digital mammography | 1992 | 50-69 | 2 | Fully implemented in 2002 | Ranging from 58·2% to 66·7% (from 1997 to 2015) | Nationwide data: 80·3% (2013-15); 74·1% (2018-20) |
| **Sweden** | Screen-film mammography and digital mammography (by 2016, 100% digital mammography) ^c^ | 1974 | 40-74 | 1.5-2 | 93% (1990); fully rolled out in 1997 | 81% (1995-1996); 73·2% (in 2013, in Stockholm and Gotland); 81% (2017-2018) | 90·4% (2013-15); 95·2% (2018-20) ^d^ |
| **Countries with implementation of mammography screening programmes after 1998** | | | | | | | |
| **Austria ^e^** | Digital mammography and ultrasound | 2007-2008 in Tyrol; 2014 nationally | 45-69 | 2 | Fully implemented in 2014 | Ages 45-69: 37% (2014-2015); 41% (2016-2017); 41% (2018-2019) | 72·7 % (2013-15); 74·7% (2018-20) |
| **Belgium (Flanders) ^f^** | Screen-film mammography and digital mammography (the latter was introduced in 2007; in 2016, 99% digital mammography) | 2001 | 50-69 | 2 | 22·0% (2002); 68·0% (2006); 88·9% (2010); 95·1% (2014); 96·0% (2016) | 48·0-57·8% (2002 to 2016) | Nationwide data: 75·5% (2013-15); 67·4% (2018-20) |
| **Belgium (Wallonia) ^f^** | Screen-film mammography and digital mammography (the latter was extensively implemented in 2008; since 2013, digital mammography is mandatory) | 2002 | 50-69 | 2 | 98·7% (2013) | 6·4-8·0% (2009 to 2017) | Nationwide data: 75·5% (2013-15); 67·4% (2018-20) |
| **Belgium (Brussels) ^f^** | Screen-film mammography and digital mammography (most mammography units switched from screen-film to digital mammography between 2008 and 2011) | 2002 | 50-69 | 2 | 74·6% (2003-2004); 97·5% (2006-2007); 97·1% (2009-2010); 93·9% (2012-2013) | 5·7% (2003-2004); 9·5% (2006-2007); 10·7% (2009-2010); 10·3% (2012-2013) | Nationwide data: 75·5% (2013-15); 67·4% (2018-20) |

**Supplementary Table 2.** Continued.

| **Country/ region** | **Test(s)** | **Year of programme initiation** | **Target ages (years)** | **Screening interval (years)** | **Invitation coverage [invitation / eligible] in organised screening (year of data collection)** | **Participation rates [participation / invited] in organised screening (year of data collection)** | **Proportion of the population aged 50-69 years who have done a mammography within the preceding 2 years ^a^** |
| --- | --- | --- | --- | --- | --- | --- | --- |
| **Czech Republic ^g^** | Screen-film mammography and digital mammography (by 2016, 100% digital mammography) | 2002 | 45-69; 45+ since 2010 | 2 | Not applicable ^g^ | Not applicable ^g^ | 76·7% (2013-15); 77·1% (2018-20) |
| **Denmark** | Screen-film mammography and digital mammography (by 2016, 100% digital mammography) | 2008 | 50-69 | 2 | 82·3% (2013) | 83·5% (2013) | 81·5% (2013-15); 82·0% (2018-20) |
| **Estonia** | Screen-film mammography and digital mammography (by 2016, 100% digital mammography) | 2003 ^h^ | 50-62 (2003-2017); 50-69 (2018 on) | 2 | 69·2% (2014, age group 50-64) | 37% (2003); 51% (2006);  66·3% (2014) | 39·0% (2013-15);  43·6% (2018-20) |
| **France (Cote d’Or)** | Screen-film mammography and digital mammography | 2004 | 50-74 | 2 | No information | Ranging from 55·6% to 61·7% between 2005-2006 and 2018-2019 | Nationwide data: 87·0% (2013-15); 70·0% (2018-20) |
| **France (Doubs)** | Screen-film mammography and digital mammography | 2003 | 50-74 | 2 | Above 90% over the period 2005-2018 (age group 50-74) | Ranging from 46·6% to 59·6% between 2005 and 2018 (age group 50-74) | Nationwide data: 87·0% (2013-15); 70·0% (2018-20) |
| **France (Herault)** | Screen-film mammography and digital mammography | 2003 | 50-74 | 2 | Above 95% over the period 2005-2018 (age group 50-74) | Ranging from 42·9% to 53·0% between 2005-2006 and 2018-2019 (age group 50-74) | Nationwide data: 87·0% (2013-15); 70·0% (2018-20) |
| **Germany ^i^** | Screen-film mammography and digital mammography (by 2016, 100% digital mammography) | 2005 (in Bremen since 2001 and in Bavaria since 2003) | 50-69 | 2 | 4·9% (2005-2006); 41·4% (2007); 78·0% (2008); 91·7% (2009); 93·4% (2010); 90·8% (2012) | 56·3% (2012) | 73·5% (2013-15); 65·7% (2018-20) |
| **Ireland** | Screen-film mammography and digital mammography (by 2016, 100% digital mammography) | 2000 | 50-64 (2000-2014); extension up to 69 starting in 2015 | 2 | ~100% (2013) | 70·2% (2013) | 66·9% (2013-15);  66·7% (2018-20) |
| **Lithuania ^j^** | Screen-film mammography and digital mammography (by 2016, 51% digital mammography) | 2005 | 50-69 | 2 | No information | No information | 46·5% (2013-15); 52·8% (2018-20) |

**Supplementary Table 2.** Continued.

| **Country/ region** | **Test(s)** | **Year of programme initiation** | **Target ages (years)** | **Screening interval (years)** | **Invitation coverage [invitation / eligible] in organised screening (year of data collection)** | **Participation rates [participation / invited] in organised screening (year of data collection)** | **Proportion of the population aged 50-69 years who have done a mammography within the preceding 2 years ^a^** |
| --- | --- | --- | --- | --- | --- | --- | --- |
| **Portugal (North)** | Screen-film mammography and digital mammography (by 2016, 100% digital mammography) | 1999 | 45-69 | 2 | 55·4% (2013) | Year 2013, 50-69-year-olds: 56·7% (North) | Nationwide data: 84·2% (2013-15); 81·1% (2018-20) |
| **Slovenia** | Screen-film mammography and digital mammography (by 2016, 100% digital mammography) | 2008 | 50-69 | 2 | 20·9% (2013); 98% (2019) | 63-94% (2008 to 2019) | 60·4% (2013-15);  67·8% (2018-20) |
| **Spain (Andalucia)** | Screen-film mammography and digital mammography | 2000 | 50-65 (2000-2007); 50-69 (2008-2016) | 2 | Fully implemented in 2008 | 65·5% (2002); 78·2% (2008); 79·8% (2016) | Nationwide data: 80·3% (2013-15); 74·1% (2018-20) |
| **Spain (Girona)** | Screen-film mammography and digital mammography | 1999 | 50-69 | 2 | Fully implemented in 2002 | 60·6% (2022) | Nationwide data: 80·3% (2013-15); 74·1% (2018-20) |
| **Switzerland (St. Gallen)** | Digital mammography | 2010 | 50-69 | 2 | 82% (average 2014-19) | 41% (2010-13) – 52% (2018-19) | 38·9% (2018-19) ^k^ |
| **Switzerland (Grisons)** | Digital mammography | 2011 | 50-69 | 2 | 86% (average 2014-19) | 32% (2010-13) – 50% (2018-19) | 40·3% (2018-19) ^k^ |

^a^ Data are from the European Health Interview Survey, 2013-15 and 2018-20 reported in Cardoso et al. 2023.^(Supplementary ref.4)^

^b^ Singe-view mammography up to 2005 and two-view mammography thereafter. In some areas of England, females ages 47 to 73 years have been invited as part of a randomised trial. Also, those older than 70 can still be screened every 3 years but are not invited.

^c^ In Sweden, in 1985, implementation of two-view mammography screening was recommended in the whole country.

^d^ Mammography use in 2013-15 refers to the preceding 3 years.

^e^ In Austria, females ages 40-44 and 70-75 years may also request participation in the screening programme. Ultrasound is additionally performed in females with dense breast tissue. Although the nationwide organised programme was implemented in 2014 only, opportunistic screening is available to females aged 40 years or older since 1974.

^f^ In Belgium, there is also ongoing opportunistic screening (without double reading). In Flanders, in 2002-2016, 15-20% of the eligible population was estimated to be covered through opportunistic screening. In Wallonia, in 2016, 47.1% of eligible population were estimated to have taken part in opportunistic screening; in Brussels, in 2011-2012, about 42.7%.

^g^ In the Czech Republic, eligible individuals are referred to screening mammography by their General Practitioners or gynaecologists. If further assessment is deemed necessary, these are provided during the screening visit at the screening centre. The proportion of the eligible population taking part in opportunistic screening was estimated to be >15% (close to 25% in some regions) in 2002; however, it declined to under 2% in 2022. Invited are only those not attending mammography during last 3 years. Three-year examination coverage by screening mammography among the age group 45-69 was 66% in 2022, according to the data from the National Registry of Reimbursed Health Services.

^h^ In Estonia, fully organized screening programme based on invitations started in 2004.

^i^ In Germany, opportunistic screening has been offered since the 1980s (in 2015, examination coverage in opportunistic screening was estimated to be 5·0%).

^j^ In Lithuania, there is no centrally organised invitation system, and the eligible population are referred by their General Practitioners.

^k^ For Switzerland (St. Gallen and Grisons), data on proportion of the population aged 50-69 years who have done a mammography within the preceding 2 years were provided by the contact person at the cancer registry.

**Supplementary Table 3.** Average annual percent changes (AAPCs) in age-standardised incidence of invasive breast cancer for the 10-year period preceding screening implementation (“Before”) and for the 10-year period starting from the year prior to screening implementation (“After”), by age at diagnosis.

| **Country/ region** | **Time period** ^a^ | **All ages** | **0-49** | **50-69** | **70-79** | **80+** |
| --- | --- | --- | --- | --- | --- | --- |
| **Countries with implementation of mammography screening programmes before 1998** | | | | | | |
| Finland | Before (1978-1986) | 3·26 (2·40 to 4·12) | 3·79 (2·18 to 5·42) | 2·99 (1·74 to 4·25) | 2·64 (0·71 to 4·61) | 4·46 (1·05 to 7·98) |
|  | After (1986-1995) | 2·04 (1·56 to 2·53) | 1·74 (0·55 to 2·94) | 2·96 (1·43 to 4·50) | 0·02 (-1·22 to 1·28) | 0·58 (-1·36 to 2·56) |
| Netherlands | Before (no data) | - | - | - | - | - |
|  | After (1989-1997) ^b^ | 2·18 (0·76 to 3·61) | 0·72 (-0·10 to 1·55) | 3·63 (1·40 to 5·91) | 0·18 (-1·09 to 1·47) | 0·96 (-0·73 to 2·68) |
| Norway | Before (1985-1994) | 0·81 (-0·12 to 1·76) | 0·90 (-0·41 to 2·23) | 1·34 (-0·28 to 2·99) | 0·09 (-0·80 to 0·98) | -1·26 (-2·94 to 0·44) |
|  | After (1994-2003) | 2·86 (1·77 to 3·96) | 0·33 (-0·47 to 1·13) | 5·31 (3·25 to 7·42) | -1·23 (-2·59 to 0·15) | -0·48 (-1·38 to 0·43) |
| **Countries with implementation of mammography screening programme after 1998** | | | | | | |
| Austria | Before (2004-2013) | 0·22 (-0·42 to 0·96) | 0·95 (-0·08 to 1·98) | -0·31 (-1·16 to 0·55) | 1·11 (0·04 to 2·18) | -0·75 (-2·00 to 0·53) |
|  | After (2013-2018) | -1·61 (-2·85 to -0·35) | -2·40 (-3·46 to -1·34) | -2·30 (-4·93 to 0·40) | 0·82 (-1·87 to 3·58) | 1·25 (-1·91 to 4·52) |
| Belgium (Flanders) | Before (no data) | - | - | - | - | - |
|  | After (2001-2009) | -0·82 (-1·43 to -0·20) | 0·14 (-1·02 to 1·31) | -1·89 (-2·72 to -1·06) | 0·88 (-0·29 to 2·07) | 2·43 (1·50 to 3·36) |
| Belgium (Wallonia and Brussels) | Before (no data) | - | - | - | - | - |
|  | After (2004-2010) | -0·70 (-1·82 to 0·42) | -1·71 (-3·25 to -0·16) | -0·94 (-2·91 to 1·06) | 1·70 (0·62 to 2·78) | 1·56 (-1·31 to 4·50) |
| Czech Republic | Before (1992-2001) | 1·67 (0·91 to 2·45) | 0·42 (-0·93 to 1·80) | 2·28 (1·66 to 2·90) | 1·45 (0·09 to 2·83) | 1·79 (0·44 to 3·15) |
|  | After (2001-2010) | 0·97 (-0·25 to 2·21) | 0·80 (0·04 to 1·57) | 0·95 (-0·64 to 2·55) | 1·65 (-0·32 to 3·65) | 0·18 (-1·43 to 1·82) |
| Denmark | Before (1998-2007) | 0·52 (-0·09 to 1·12) | -0·15 (-1·15 to 0·85) | 0·53 (-0·20 to 1·26) | 1·22 (0·47 to 1·97) | 1·11 (-0·36 to 2·60) |
|  | After (2007-2016) | -1·06 (-3·43 to 1·36) | 1·56 (0·47 to 2·65) | -2·33 (-5·92 to 1·41) | -0·42 (-1·47 to 0·65) | 1·20 (-0·11 to 2·53) |
| Estonia | Before (1995-2002) | 1·59 (-0·13 to 3·34) | 0·15 (-4·40 to 4·92) | 1·91 (-0·05 to 3·91) | 2·87 (-1·09 to 6·98) | 3·03 (-2·22 to 8·55) |
|  | After (2002-2011) | 1·45 (-0·15 to 3·09) | 1·14 (-1·35 to 3·69) | 1·29 (-1·08 to 3·71) | 3·09 (0·37 to 5·88) | 1·37 (-1·96 to 4·81) |
| Germany (6 states) | Before (no data) | - | - | - | - | - |
|  | After (2004-2013) | 0·83 (-0·46 to 2·12) | 1·16 (0·73 to 1·58) | 1·03 (-1·12 to 3·22) | 0·43 (-1·19 to 1·05) | -1·18 (-1·81 to -0·54) |
| Germany (Bavaria) | Before (no data) | - | - | - | - | - |
|  | After (2002-2011) | 0·74 (-0·14 to 1·63) | 0·77 (0·28 to 1·26) | 0·93 (-0·37 to 2·25) | 0·72 (0·16 to 1·29) | -0·79 (-1·91 to 0·35) |
| Ireland | Before (1994-1999) | 1·12 (-0·17 to 2·43) | 1·11 (-0·31 to 2·56) | 1·34 (-1·61 to 4·38) | -0·39 (-3·88 to 3·22) | 3·36 (-3·66 to 10·88) |
|  | After (1999-2008) | 1·89 (0·50 to 3·30) | 1·88 (0·55 to 3·23) | 1·72 (-0·46 to 3·95) | 2·48 (1·05 to 3·94) | 2·35 (0·91 to 3·80) |
| Lithuania | Before (1995-2004) | 1·68 (0·35 to 3·03) | 0·27 (-1·99 to 2·58) | 1·68 (0·61 to 2·77) | 4·37 (0·88 to 7·97) | 3·38 (1·26 to 5·55) |
|  | After (2004-2013) | 2·07 (1·25 to 2·90) | 1·97 (1·01 to 2·94) | 2·45 (0·85 to 4·08) | 0·93 (-0·22 to 2·09) | 1·43 (-1·58 to 4·54) |
| Slovenia | Before (1998-2007) | 0·94 (0·23 to 1·65) | -0·05 (-0·95 to 0·86) | 1·51 (0·32 to 2·72) | 0·30 (-1·35 to 1·97) | 1·76 (0·05 to 3·49) |
|  | After (2007-2016) | 1·00 (-0·11 to 2·13) | 2·06 (-0·20 to 4·38) | 0·58 (-0·67 to 1·85) | 1·78 (-0·10 to 3·69) | -1·60 (-2·96 to -0·22) |

AAPC, average annual percentage change.

^a^ If data were available for at least a 6-year period (out of the 10-year period), AAPCs for such time periods were also calculated. If data for such time periods were not available, AAPCs were not calculated (marked as “-“). No data for England and Sweden were therefore included here. In Sweden, screening was introduced in 1974; given that our study considers data available from 1978 only, AAPCs for periods right before and right after screening implementation were not available for calculation. Furthermore, Bulgaria and Ukraine were not included here because screening had not yet been implemented or only small-scale pilot programmes were available. ^b^ Netherlands: Screening was extended from ages 50-69 to 50-74 in 1998.

**Supplementary Table 4.** Average annual percent changes (AAPCs) in age-standardised incidence of in situ breast cancer for the 10-year period preceding screening implementation (“Before”) and for the 10-year period starting from the year prior to screening implementation (“After”), by age.

| **Country/ region** | **Time period** ^a^ | **All ages** | **0-49** | **50-69** | **70-79** | **80+** |
| --- | --- | --- | --- | --- | --- | --- |
| **Countries with implementation of mammography screening programmes before 1998** | | | | | | |
| Netherlands | Before (no data) | - | - | - | - | - |
|  | After (1989-1997) ^b^ | 12·03 (7·40 to 16·86) | 4·98 (1·63 to 8·44) | 16·74 (9·82 to 24·08) | 2·61 (0·42 to 4·84) | 9·86 (4·60 to 15·40) |
| Norway | Before (no data) | - | - | - | - | - |
|  | After (1998-2003) | 8·62 (1·01 to 16·80) | -5·13 (-15·94 to 7·08) | 13·19 (-0·02 to 28·14) | 0·46 (-23·79 to 32·42) | -7·49 (-30·96 to 23·96) |
| **Countries with implementation of mammography screening after 1998** | | | | | | |
| Austria | Before (2004-2013) | 3·40 (1·41 to 5·44) | 4·14 (0·51 to 7·92) | 2·76 (1·02 to 4·54) | 4·95 (1·04 to 9·00) | 1·32 (-3·46 to 6·34) |
|  | After (2013-2018) | 0·31 (-4·10 to 4·93) | -0·99 (-7·06 to 5·46) | 0·50 (-4·41 to 5·66) | 2·04 (-8·19 to 13·41) | 4·39 (-10·93 to 22·36) |
| Belgium (Flanders) | Before (no data) | - | - | - | - | - |
|  | After (2001-2009) | 0·61 (-1·02 to 2·26) | 1·25 (-0·92 to 3·46) | 0·02 (-2·35 to 2·44) | 3·79 (-2·67 to 10·69) | 5·74 (-5·03 to 17·73) |
| Belgium (Wallonia and Brussels) | Before (no data) | - | - | - | - | - |
|  | After (2004-2010) | 0·73 (-3·28 to 4·92) | 3·53 (-2·15 to 9·54) | -0·47 (-4·92 to 4·20) | -0·63 (-6·35 to 5·44) | 3·99 (-8·15 to 17·73) |
| Czech Republic | Before (1992-2001) | 10·60 (6·96 to 14·37) | 5·63 (0·00 to 11·57) | 16·05 (10·73 to 21·62) | 11·70 (1·84 to 22·51) | 0·77 (-14·63 to 18·95) |
|  | After (2001-2010) | 9·60 (6·30 to 13·00) | 7·04 (4·73 to 9·39) | 10·15 (5·49 to 15·00) | 16·64 (8·18 to 25·76) | 5·41 (-3·68 to 15·37) |
| Germany (6 states) | Before (no data) | - | - | - | - | - |
|  | After (2004-2013) | 8·32 (3·09 to 13·81) | 2·42 (-0·19 to 5·11) | 10·79 (3·96 to 18·07) | 5·58 (2·56 to 8·69) | 6·71 (0·06 to 13·81) |
| Germany (Bavaria) | Before (no data) | - | - | - | - | - |
|  | After (2002-2011) | 9·67 (5·61 to 13·90) | 5·36 (1·40 to 9·47) | 11·48 (6·95 to 16·21) | 6·71 (3·45 to 10·07) | 12·04 (5·60 to 18·87) |
| Ireland | Before (1994-1999) | 12·04 (2·83 to 22·09) | 13·55 (4·12 to 23·82) | 9·95 (4·00 to 16·24) | 38·74 (-13·25 to 121·88) | 0·17 (-36·74 to 58·59) |
|  | After (1999-2008) | 9·70 (6·33 to 13·16) | 8·37 (1·64 to 15·55) | 10·21 (5·35 to 15·28) | 12·31 (6·72 to 18·20) | 22·77 (11·33 to 35·38) |
| Slovenia | Before (1998-2007) | - | - | - | - | - |
|  | After (2007-2016) | 3·75 (1·01 to 6·56) | 3·44 (-0·03 to 7·02) | 3·62 (-0·17 to 7·56) | 4·80 (-1·22 to 11·19) | 4·62 (-2·19 to 11·89) |

^a^ If data were available for at least a 6-year period (out of the 10-year period), AAPCs for such time periods were also shown. If data for such time periods were not available, AAPCs were not calculated (“marked as “-“).

^b^ Netherlands: Screening was extended from ages 50-69 to 50-74 in 1998.

**Supplementary Table 5.** Average annual percent changes (AAPCs) in age-standardised incidence of stage I breast cancer for the 10-year period preceding screening implementation (“Before”) and for the 10-year period starting from the year prior to screening implementation (“After”), by age.

| **Country/ region** | **Time period** ^a^ | **All ages** | **0-49** | **50-69** | **70-79** | **80+** |
| --- | --- | --- | --- | --- | --- | --- |
| **Countries with implementation of mammography screening programmes before 1998** | | | | | | |
| Netherlands | Before (no data) | - | - | - | - | - |
|  | After (1989-1997) ^b^ | 6·01 (3·43 to 8·65) | 1·37 (0·41 to 2·34) | 9·69 (5·58 to 13·95) | 1·07 (-0·85 to 3·02) | 0·29 (-2·27 to 2·92) |
| Norway | Before (no data) | - | - | - | - | - |
|  | After (1998-2003) | 3·84 (0·98 to 6·78) | -1·38 (-4·23 to 1·55) | 6·80 (2·83 to 10·92) | -4·54 (-11·64 to 3·15) | 0·83 (-5·06 to 7·08) |
| **Countries with implementation of mammography screening after 1998** | | | | | | |
| Austria | Before (2004-2013) | ^c^ | ^c^ | ^c^ | ^c^ | ^c^ |
|  | After (2013-2018) | ^c^ | ^c^ | ^c^ | ^c^ | ^c^ |
| Belgium (Flanders) | Before (no data) | - | - | - | - | - |
|  | After (2001-2009) | -0·31 (-0·91 to 0·29) | 0·74 (-0·69 to 2·20) | -1·25 (-2·28 to -0·21) | 2·15 (-0·25 to 4·09) | 3·65 (-0·25 to 7·70) |
| Belgium (Wallonia and Brussels) | Before (no data) | - | - | - | - | - |
|  | After (2004-2010) | 0·20 (-1·85 to 2·30) | -0·80 (-4·71 to 3·28) | 0·11 (-2·52 to 2·80) | 2·84 (0·59 to 5·13) | 0·99 (-2·92 to 5·05) |
| Czech Republic | Before (1992-2001) | 9·68 (8·16 to 11·22) | 5·89 (3·13 to 8·73) | 11·94 (10·31 to 13·59) | 9·09 (7·40 to 10·82) | 8·24 (5·67 to 10·87) |
|  | After (2001-2010) | 4·65 (2·44 to 6·91) | 2·40 (0·68 to 4·16) | 5·30 (2·71 to 7·97) | 6·12 (2·28 to 10·11) | 2·02 (-0·97 to 5·10) |
| Germany (6 states) | Before (no data) | - | - | - | - | - |
|  | After (2004 to 2013) | 3·32 (1·16 to 5·52) | 0·58 (0·02 to 1·13) | 4·51 (1·36 to 7·75) | 2·68 (1·78 to 3·58) | 2·20 (0·46 to 3·97) |
| Germany (Bavaria) | Before (no data) | - | - | - | - | - |
|  | After (2002 to 2011) | 3·68 (2·04 to 5·35) | 1·72 (0·43 to 3·03) | 4·52 (2·30 to 6·79) | 2·91 (2·27 to 3·56) | 5·34 (2·86 to 7·88) |
| Ireland | Before (1994-1999) | 2·94 (1·09 to 4·82) | 2·27 (-2·08 to 6·81) | 3·93 (1·32 to 6·61) | -1·12 (-9·52 to 8·07) | 7·24 (-1·63 to 16·92) |
|  | After (1999-2008) | 5·03 (3·01 to 7·09) | 3·47 (2·60 to 4·34) | 5·56 (2·22 to 9·01) | 6·07 (3·42 to 8·78) | 3·15 (-1·87 to 8·42) |
| Lithuania | Before (1995-2004) | 12·63 (9·13 to 16·24) | 9·63 (3·41 to 16·22) | 13·75 (10·70 to 16·88) | 17·91 (11·54 to 24·64) | 17·27 (7·77 to 27·61) |
|  | After (2004-2013) | 6·86 (4·86 to 8·91) | 5·31 (3·47 to 7·17) | 8·03 (4·73 to 11·43) | 5·03 (1·79 to 8·37) | 3·83 (-0·80 to 8·68) |
| Slovenia | Before (1998-2007) | - | - | - | - | - |
|  | After (2007-2016) | 2·17 (0·92 to 3·42) | 2·13 (-0·43 to 4·77) | 2·30 (1·12 to 3·50) | 1·69 (-0·97 to 4·42) | 1·76 (-4·77 to 8·75) |

^a^ If data were available for at least a 6-year period (out of the 10-year period), AAPCs for such time periods were also shown. If data for such time periods were not available, AAPCs were not calculated (“marked as “-“).

^b^ Netherlands: Screening was extended from ages 50-69 to 50-74 in 1998.

^c^ Austria: Before screening implementation – Localised, All ages: 1·95 (1·16 to 2·73); 0-49: 1·95 (0·35 to 3·57); 50-69: 1·78 (0·94 to 2·63); 70-79: 2·75 (1·53 to 3·99); 80+: 1·14 (-0·35 to 2·64). Regional, All ages: -1·89 (-3·81 to 0·07); 0-49: 0·17 (-1·76 to 2·15); 50-69: -3·03 (-5·13 to -0·89); 70-79: -1·38 (-3·22 to 0·49); 80+: -2·89 (-5·43 to -0·29).

After screening implementation – Localised, All ages: -1·71 (-3·44 to 0·06); 0-49: -3·26 (-4·87 to -1·63); 50-69: -2·10 (-5·10 to 0·99); 70-79: 1·09 (-1·98 to 4·25); 80+: 1·58 (-3·86 to 7·33). Regional, All ages: -0·79 (-2·23 to 0·68); 0-49: -0·63 (-2·71 to 1·50); 50-69: -1·84 (-4·94 to 1·36); 70-79: 0·55 (-2·61 to 3·82); 80+: 2·75 (-0·40 to 6·00).

**Supplementary Table 6.** Average annual percent changes (AAPCs) in age-standardised incidence of stage II breast cancer for the 10-year period preceding screening implementation (“Before”) and for the 10-year period starting from the year prior to screening implementation (“After”), by age.

| **Country/ region** | **Time period ^a^** | **All ages** | **0-49** | **50-69** | **70-79** | **80+** |
| --- | --- | --- | --- | --- | --- | --- |
| **Countries with implementation of mammography screening programmes before 1998** | | | | | | |
| Netherlands | Before (no data) | - | - | - | - | - |
|  | After (1989-1997) ^b^ | 0·59 (-0·52 to 1·72) | 0·81 (-0·38 to 2·01) | 0·85 (-1·03 to 2·75) | -0·81 (-2·38 to 0·78) | 0·98 (-1·15 to 3·15) |
| Norway | Before (no data) | - | - | - | - | - |
|  | After (1998-2003) | 2·63 (0·70 to 4·59) | 1·99 (-2·58 to 6·77) | 4·03 (0·86 to 7·29) | -1·20 (-5·41 to 3·19) | 0·60 (-1·25 to 2·49) |
| **Countries with implementation of mammography screening after 1998** | | | | | | |
| Austria | Before (2004-2013) | ^c^ | ^c^ | ^c^ | ^c^ | ^c^ |
|  | After (2013-2018) | ^c^ | ^c^ | ^c^ | ^c^ | ^c^ |
| Belgium (Flanders) | Before (no data) | - | - | - | - | - |
|  | After (2001-2009) | -1·94 (-3·08 to -0·79) | -1·21 (-2·92 to 0·53) | -3·41 (-4·82 to -1·99) | 0·23 (-1·39 to 1·88) | 3·79 (1·68 to 5·95) |
| Belgium (Wallonia and Brussels) | Before (no data) | - | - | - | - | - |
|  | After (2004-2010) | -2·21 (-3·31 to -1·10) | -3·24 (-6·05 to -0·35) | -2·73 (-4·58 to -0·84) | 0·55 (-2·02 to 3·20) | 1·86 (-1·82 to 5·68) |
| Czech Republic | Before (1992-2001) | 0·74 (-0·38 to 1·87) | -0·70 (-2·26 to 0·89) | 1·17 (-0·14 to 2·50) | 0·90 (-0·66 to 2·47) | 2·27 (0·51 to 4·06) |
|  | After (2001-2010) | -2·04 (-3·14 to -0·92) | -1·34 (-2·78 to 0·12) | -2·85 (-4·45 to -1·24) | -1·32 (-3·04 to 0·44) | -0·15 (-1·91 to 1·65) |
| Germany (6 states) | Before (no data) | - | - | - | - | - |
|  | After (2004 to 2013) | 1·38 (0·24 to 2·53) | 2·49 (1·91 to 3·07) | 0·60 (-1·30 to 2·54) | 1·82 (1·08 to 2·58) | 2·32 (1·53 to 3·13) |
| Germany (Bavaria) | Before (no data) | - | - | - | - | - |
|  | After (2002 to 2011) | 0·79 (-0·06 to 1·65) | 0·79 (-0·23 to 1·83) | -0·22 (-1·23 to 0·80) | 2·91 (1·83 to 4·00) | 6·07 (4·50 to 7·66) |
| Ireland | Before (1994-1999) | 1·55 (-0·70 to 3·84) | 0·43 (-3·37 to 4·38) | 2·18 (-3·56 to 8·27) | 0·75 (-4·14 to 5·89) | 4·65 (-8·75 to 20·01) |
|  | After (1999-2008) | -0·25 (-1·75 to 1·28) | 0·31 (-0·89 to 1·53) | -0·98 (-3·27 to 1·37) | 0·90 (-0·66 to 2·48) | 1·67 (0·03 to 3·33) |
| Lithuania | Before (1995-2004) | 2·22 (0·92 to 3·54) | 0·02 (-1·84 to 1·92) | 3·15 (1·89 to 4·43) | 3·80 (-0·78 to 8·59) | 5·18 (0·70 to 9·87) |
|  | After (2004-2013) | -0·55 (-1·59 to 0·51) | -1·14 (-3·74 to 1·52) | -0·39 (-2·71 to 1·99) | -0·25 (-3·24 to 2·84) | 0·97 (-2·54 to 4·60) |
| Slovenia | Before (1998-2007) | - | - | - | - | - |
|  | After (2007-2016) | 1·17 (-0·68 to 3·06) | 2·65 (-0·26 to 5·65) | -0·05 (-2·18 to 2·12) | 3·48 (0·30 to 6·75) | -0·73 (-3·21 to 1·80) |

^a^ If data were available for at least a 6-year period (out of the 10-year period), AAPCs for such time periods were also shown. If data for such time periods were not available, AAPCs were not calculated (“marked as “-“).

^b^ Netherlands: Screening was extended from ages 50-69 to 50-74 in 1998.

^c^ Austria: Before screening implementation – Localised, All ages: 1·95 (1·16 to 2·73); 0-49: 1·95 (0·35 to 3·57); 50-69: 1·78 (0·94 to 2·63); 70-79: 2·75 (1·53 to 3·99); 80+: 1·14 (-0·35 to 2·64). Regional, All ages: -1·89 (-3·81 to 0·07); 0-49: 0·17 (-1·76 to 2·15); 50-69: -3·03 (-5·13 to -0·89); 70-79: -1·38 (-3·22 to 0·49); 80+: -2·89 (-5·43 to -0·29).

After screening implementation – Localised, All ages: -1·71 (-3·44 to 0·06); 0-49: -3·26 (-4·87 to -1·63); 50-69: -2·10 (-5·10 to 0·99); 70-79: 1·09 (-1·98 to 4·25); 80+: 1·58 (-3·86 to 7·33). Regional, All ages: -0·79 (-2·23 to 0·68); 0-49: -0·63 (-2·71 to 1·50); 50-69: -1·84 (-4·94 to 1·36); 70-79: 0·55 (-2·61 to 3·82); 80+: 2·75 (-0·40 to 6·00).

**Supplementary Table 7.** Average annual percent changes (AAPCs) in age-standardised incidence of stage III breast cancer for the 10-year period preceding screening implementation (“Before”) and for the 10-year period starting from the year prior to screening implementation (“After”), by age.

| **Country/ region** | **Time period** ^a^ | **All ages** | **0-49** | **50-69** | **70-79** | **80+** |
| --- | --- | --- | --- | --- | --- | --- |
| **Countries with implementation of mammography screening programmes before 1998** | | | | | | |
| Netherlands | Before (no data) | - | - | - | - | - |
|  | After (1989-1997) ^b^ | -1·73 (-2·63 to -0·82) | -1·85 (-4·18 to 0·53) | -3·48 (-4·73 to -2·22) | 1·01 (-0·16 to 2·20) | 1·09 (-2·04 to 4·32) |
| Norway | Before (no data) | - | - | - | - | - |
|  | After (1998-2003) | 0·57 (-3·32 to 4·61) | 6·46 (-4·53 to 18·71) | -0·36 (-2·07 to 1·37) | -3·02 (-8·26 to 2·53) | -3·20 (-10·27 to 4·42) |
| **Countries with implementation of mammography screening after 1998** | | | | | | |
| Austria | Before (2004-2013) | ^c^ | ^c^ | ^c^ | ^c^ | ^c^ |
|  | After (2013-2018) | ^c^ | ^c^ | ^c^ | ^c^ | ^c^ |
| Belgium (Flanders) | Before (no data) | - | - | - | - | - |
|  | After (2001-2009) | -4·30 (-5·74 to -2·84) | -3·64 (-5·52 to -1·72) | -4·80 (-6·76 to -2·81) | -4·82 (-7·00 to -2·60) | -2·23 (-5·21 to 0·85) |
| Belgium (Wallonia and Brussels) | Before (no data) | - | - | - | - | - |
|  | After (2004-2010) | 1·17 (-5·12 to 7·86) | -0·42 (-8·11 to 7·92) | 0·71 (-7·03 to 9·09) | 3·61 (-3·48 to 11·22) | 2·03 (-2·81 to 7·12) |
| Czech Republic | Before (1992-2001) | -1·58 (-3·04 to -0·10) | -1·06 (-4·50 to 2·51) | -2·65 (-4·90 to -0·35) | -0·94 (-2·85 to 1·01) | 1·24 (-1·58 to 4·14) |
|  | After (2001-2010) | -2·32 (-3·90 to -0·72) | -0·24 (-3·83 to 3·48) | -3·80 (-5·74 to -1·81) | -1·06 (-3·17 to 1·09) | -1·37 (-4·56 to 1·91) |
| Germany (6 states) | Before (no data) | - | - | - | - | - |
|  | After (2004 to 2013) | -2·21 (-3·26 to -1·14) | -0·78 (-2·17 to 0·64) | -3·30 (-4·84 to -1·72) | -1·51 (-2·78 to -0·22) | -1·23 (-2·47 to 0·03) |
| Germany (Bavaria) | Before (no data) | - | - | - | - | - |
|  | After (2004-2013) | 1·08 (-3·30 to 5·65) | 1·70 (-2·99 to 6·62) | -0·16 (-4·77 to 4·68) | 3·02 (-1·68 to 7·95) | 2·87 (-0·20 to 6·03) |
| Ireland | Before (1994-1999) | -1·10 (-8·34 to 6·71) | 8·41 (-2·81 to 20·92) | -3·60 (-13·60 to 7·56) | 1·92 (-10·29 to 15·79) | -11·33 (-21·20 to -0·22) |
|  | After (1999-2008) | 2·84 (1·35 to 4·36) | 3·51 (0·48 to 6·64) | 1·76 (-0·76 to 4·35) | 1·82 (-2·64 to 6·49) | 12·82 (5·08 to 21·13) |
| Lithuania | Before (1995-2004) | -0·79 (-2·56 to 1·02) | -4·21 (-9·63 to 1·55) | -1·75 (-4·76 to 1·35) | 4·28 (-0·86 to 9·68) | 3·81 (0·83 to 6·89) |
|  | After (2004-2013) | -4·41 (-6·40 to -2·39) | -1·44 (-5·59 to 2·89) | -5·82 (-8·81 to -2·73) | -4·36 (-9·15 to 0·68) | -2·12 (-9·11 to 5·41) |
| Slovenia | Before (1998-2007) | - | - | - | - | - |
|  | After (2007-2016) | 0·06 (-3·09 to 3·30) | -0·42 (-7·41 to 7·09) | 2·35 (-3·99 to 9·10) | -0·89 (-5·74 to 4·20) | -5·12 (-9·78 to -0·21) |

^a^ If data were available for at least a 6-year period (out of the 10-year period), AAPCs for such time periods were also shown. If data for such time periods were not available, AAPCs were not calculated (“marked as “-“).

^b^ Netherlands: Screening was extended from ages 50-69 to 50-74 in 1998.

^c^ Austria: Before screening implementation – Localised, All ages: 1·95 (1·16 to 2·73); 0-49: 1·95 (0·35 to 3·57); 50-69: 1·78 (0·94 to 2·63); 70-79: 2·75 (1·53 to 3·99); 80+: 1·14 (-0·35 to 2·64). Regional, All ages: -1·89 (-3·81 to 0·07); 0-49: 0·17 (-1·76 to 2·15); 50-69: -3·03 (-5·13 to -0·89); 70-79: -1·38 (-3·22 to 0·49); 80+: -2·89 (-5·43 to -0·29).

After screening implementation – Localised, All ages: -1·71 (-3·44 to 0·06); 0-49: -3·26 (-4·87 to -1·63); 50-69: -2·10 (-5·10 to 0·99); 70-79: 1·09 (-1·98 to 4·25); 80+: 1·58 (-3·86 to 7·33). Regional, All ages: -0·79 (-2·23 to 0·68); 0-49: -0·63 (-2·71 to 1·50); 50-69: -1·84 (-4·94 to 1·36); 70-79: 0·55 (-2·61 to 3·82); 80+: 2·75 (-0·40 to 6·00).

**Supplementary Table 8.** Average annual percent changes (AAPCs) in age-standardised incidence of stage IV breast cancer for the 10-year period preceding screening implementation (“Before”) and for the 10-year period starting from the year prior to screening implementation (“After”), by age.

| **Country/ region** | **Time period** ^a^ | **All ages** | **0-49** | **50-69** | **70-79** | **80+** |
| --- | --- | --- | --- | --- | --- | --- |
| **Countries with implementation of mammography screening programmes before 1998** | | | | | | |
| Netherlands | Before (no data) | - | - | - | - | - |
|  | After (1989-1997) ^b^ | -0·40 (-2·17 to 1·40) | -0·98 (-4·30 to 2·45) | -1·97 (-4·25 to 0·36) | 2·57 (0·65 to 4·53) | 2·63 (1·21 to 4·06) |
| Norway | Before (no data) | - | - | - | - | - |
|  | After (1998-2003) | -5·34 (-10·01 to -0·43) | 0·85 (-12·27 to 15·94) | -8·81 (-14·71 to -2·51) | -3·09 (-8·14 to 2·24) | -4·42 (-8·09 to -0·61) |
| **Countries with implementation of mammography screening after 1998** | | | | | | |
| Austria | Before (2004-2013) | -3·71 (-6·40 to -0·94) | -3·99 (-9·50 to 1·87) | -4·76 (-7·57 to -1·88) | -1·00 (-3·64 to 1·72) | -2·34 (-3·84 to -0·82) |
|  | After (2013-2018) | -4·76 (-6·44 to -3·05) | -3·93 (-9·66 to 2·16) | -6·66 (-10·90 to -2·22) | -0·79 (-9·34 to 8·57) | -5·91 (-9·75 to -1·90) |
| Belgium (Flanders) | Before (no data) | - | - | - | - | - |
|  | After (2001-2009) | -4·30 (-5·74 to -2·84) | -3·64 (-5·52 to -1·72) | -4·80 (-6·76 to -2·81) | -4·82 (-7·00 to -2·60) | -2·23 (-5·21 to 0·85) |
| Belgium (Wallonia and Brussels) | Before (no data) | - | - | - | - | - |
|  | After (2004-2010) | 1·17 (-5·12 to 7·86) | -0·42 (-8·11 to 7·92) | 0·71 (-7·03 to 9·09) | 3·61 (-3·48 to 11·22) | 2·03 (-2·81 to 7·12) |
| Czech Republic | Before (1992-2001) | -1·58 (-3·04 to -0·10) | -1·06 (-4·50 to 2·51) | -2·65 (-4·90 to -0·35) | -0·94 (-2·85 to 1·01) | 1·24 (-1·58 to 4·14) |
|  | After (2001-2010) | -2·32 (-3·90 to -0·72) | -0·24 (-3·83 to 3·48) | -3·80 (-5·74 to -1·81) | -1·06 (-3·17 to 1·09) | -1·37 (-4·56 to 1·91) |
| Germany (6 states) | Before (no data) | - | - | - | - | - |
|  | After (2004-2013) | -2·04 (-2·82 to -1·25) | -1·40 (-0·09 to 2·91) | -2·97 (-4·52 to -1·40) | -2·17 (-3·27 to -1·06) | -3·19 (-4·22 to -2·16) |
| Germany (Bavaria) | Before (no data) | - | - | - | - | - |
|  | After (2002-2011) | -1·27 (-2·69 to 0·17) | -1·01 (-2·53 to 0·53) | -1·68 (-3·09 to -0·24) | -0·21 (-2·32 to 1·94) | -1·91 (-5·46 to 1·77) |
| Ireland | Before (1994-1999) | -1·10 (-8·34 to 6·71) | 8·41 (-2·81 to 20·92) | -3·60 (-13·60 to 7·56) | 1·92 (-10·29 to 15.79) | -11·33 (-21·20 to -0·22) |
|  | After (1999-2008) | 2·84 (1·35 to 4·36) | 3·51 (0·48 to 6·64) | 1·76 (-0·76 to 4·35) | 1·82 (-2·64 to 6·49) | 12·82 (5·08 to 21·13) |
| Lithuania | Before (1995-2004) | -0·79 (-2·56 to 1·02) | -4·21 (-9·63 to 1·55) | -1·75 (-4·76 to 1·35) | 4·28 (-0·86 to 9·68) | 3·81 (0·83 to 6·89) |
|  | After (2004-2013) | -4·41 (-6·40 to -2·39) | -1·44 (-5·59 to 2·89) | -5·82 (-8·81 to -2·73) | -4·36 (-9·15 to 0·68) | -2·12 (-9·11 to 5·41) |
| Slovenia | Before (1998-2007) | - | - | - | - | - |
|  | After (2007-2016) | 0·06 (-3·09 to 3·30) | -0·42 (-7·41 to 7·09) | 2·35 (-3·99 to 9·10) | -0·89 (-5·74 to 4·20) | -5·12 (-9·78 to -0·21) |

^a^ If data were available for at least a 6-year period (out of the 10-year period), AAPCs for such time periods were also shown. If data for such time periods were not available, AAPCs were not calculated (“marked as “-“).

^b^ Netherlands: Screening was extended from ages 50-69 to 50-74 in 1998.

**Supplementary Table 9.** Average annual percent changes (AAPCs) in age-standardised breast cancer mortality for the 10-year period preceding screening implementation (“Before”) and for the 10-year period starting from the year prior to screening implementation (“After”), by age.

| **Country/ region** | **Time period** ^a^ | **All ages** | **0-49** | **50-69** | **70-79** | **80+** |
| --- | --- | --- | --- | --- | --- | --- |
| **Countries with implementation of mammography screening programmes before 1998** | | | | | | |
| Finland | Before (1978-1986) | 1·25 (0·12 to 2·39) | -1·87 (-3·81 to 0·10) | 1·49 (-0·89 to 3·93) | 2·94 (0·82 to 5·10) | 2·42 (-0·20 to 5·10) |
|  | After (1986-1995) | -0·42 (-1·32 to 0·50) | 0·11 (-2·12 to 2·40) | -0·27 (-1·39 to 0·86) | -1·27 (-2·79 to 0·26) | -0·39 (-3·06 to 2·36) |
| Netherlands | Before (no data) | - | - | - | - | - |
|  | After (1989-1997) ^b^ | -0·81 (-1·29 to -0·32) | -1·29 (-2·89 to 0·34) | -0·81 (-1·60 to -0·02) | -0·33 (-1·54 to 0·89) | -0·86 (-2·51 to 0·81) |
| Norway | Before (1985-1994) | 0·35 (-0·60 to 1·31) | 1·39 (-0·85 to 3·68) | 0·02 (-1·37 to 1·43) | -0·07 (-2·14 to 2·04) | 0·95 (-0·58 to 2·51) |
|  | After (1994-2003) | -2·44 (-3·25 to -1·62) | -5·41 (-8·44 to -2·29) | -1·90 (-3·00 to -0·78) | -2·03 (-3·41 to -0·64) | -1·50 (-3·42 to 0·46) |
| **Countries with implementation of mammography screening after 1998** | | | | | | |
| Austria | Before (2004-2013) | -2·20 (-2·82 to -1·57) | -0·92 (-2·44 to 0·63) | -4·06 (-4·55 to -3·57) | -0·15 (-2·14 to 1·87) | -0·62 (-1·79 to 0·58) |
|  | After (2013-2018) | -0·58 (-1·20 to 0·03) | 1·27 (-1·00 to 3·60) | -1·31 (-2·96 to 0·37) | -0·83 (-3·28 to 1·69) | 0·03 (-2·01 to 2·12) |
| Belgium (Flanders) | Before (no data) | - | - | - | - | - |
|  | After (2004-2009) | -1·67 (-2·61 to -0·73) | -4·59 (-11·45 to 2·79) | -1·78 (-3·86 to 0·34) | 0·39 (-3·36 to 4·28) | -1·30 (-5·42 to 3·01) |
| Belgium (Wallonia and Brussels) | Before (no data) | - | - | - | - | - |
|  | After (2004-2010) | -1·61 (-3·88 to 0·71) | -4·56 (-9·98 to 1·19) | -1·81 (-4·59 to 1·05) | -0·49 (-3·10 to 2·20) | -0·03 (-3·14 to 3·19) |
| Czech Republic | Before (1992-2001) | -0·85 (-1·66 to -0·03) | -5·10 (-6·23 to -3·96) | -1·01 (-2·21 to 0·20) | -0·06 (-1·24 to 1·13) | 1·90 (0·16 to 3·67) |
|  | After (2001-2010) | -2·55 (-3·25 to -1·85) | -3·17 (-4·75 to -1·56) | -3·38 (-4·35 to -2·41) | -1·60 (-2·54 to -0·65) | -1·14 (-2·57 to 0·32) |
| Denmark | Before (1998-2007) | -2·54 (-3·15 to -1·93) | -6·38 (-7·70 to -5·05) | -3·20 (-4·41 to -1·98) | -0·06 (-1·15 to 1·05) | -0·96 (-2·21 to 0·31) |
|  | After (2007-2016) | -3·14 (-3·97 to -2·30) | -2·91 (-5·93 to 0·19) | -4·73 (-6·02 to -3·41) | -2·87 (-4·33 to -1·38) | 0·25 (-1·11 to 1·63) |
| Estonia | Before (1995-2002) | 0·08 (-2·15 to 2·36) | -3·23 (-12·56 to 7·09) | -0·09 (-2·08 to 1·94) | 2·24 (-2·27 to 6·95) | 2·51 (-2·40 to 7·68) |
|  | After (2002-2011) | -1·60 (-2·94 to -0·24) | -1·57 (-6·90 to 4·06) | -2·78 (-5·06 to -0·43) | -0·16 (-2·12 to 1·83) | 1·77 (-0·56 to 4·14) |
| Germany (6 states) | Before (1995-2004) | -1·74 (-2·30 to -1·17) | -4·14 (-5·51 to -2·74) | -1·50 (-2·16 to -0·84) | -1·27 (-1·98 to -0·55) | -0·55 (-1·23 to 0·13) |
|  | After (2004-2013) | -1·19 (-1·69 to -0·68) | -2·76 (-4·23 to -1·27) | -2·29 (-2·81 to -1·78) | 0·14 (-0·51 to 0·80) | 0·54 (-0·38 to 1·48) |
| Germany (Bavaria) | Before (1993-2002) | -1·58 (-2·35 to -0·81) | -4·04 (-6·40 to -1·62) | -1·42 (-2·44 to -0·39) | -1·37 (-2·16 to -0·58) | 0·28 (-0·69 to 1·26) |
|  | After (2002-2011) | -1·81 (-2·53 to -1·09) | -2·46 (-4·66 to -0·22) | -2·25 (-3·25 to -1·24) | -0·85 (-1·42 to -0·27) | -1·13 (-2·61 to 0·37) |
| Ireland | Before (1994-1999) | -2·30 (-4·60 to 0·06) | -0·73 (-9·30 to 8·66) | -2·16 (-4·72 to 0·47) | -4·13 (-8·42 to 0·35) | -2·52 (-5·10 to 0·13) |
|  | After (1999-2008) | -1·84 (-3·27 to -0·40) | -3·49 (-8·08 to 1·33) | -2·73 (-4·24 to -1·20) | -0·57 (-2·88 to 1·78) | 1·84 (-0·40 to 4·12) |
| Lithuania | Before (1995-2004) | -0·34 (-1·56 to 0·90) | -2·60 (-4·85 to -0·29) | -0·57 (-1·74 to 0·61) | 2·47 (-0·05 to 5·06) | 0·88 (-3·35 to 5·29) |
|  | After (2004-2013) | -0·58 (-1·90 to 0·77) | -1·73 (-5·73 to 2·45) | -0·48 (-2·13 to 1·20) | -0·53 (-3·37 to 2·39) | 0·97 (-3·97 to 6·16) |
| Slovenia | Before (1998-2007) | -2·03 (-3·17 to -0·88) | -5·07 (-8·00 to -2·04) | -3·09 (-5·37 to -0·75) | -0·96 (-3·42 to 1·57) | 2·07 (-1·28 to 5·54) |
|  | After (2007-2016) | -2·66 (-3·95 to -1·36) | -1·29 (-4·44 to 1·98) | -2·96 (-5·52 to -0·33) | -3·07 (-4·50 to -1·62) | -2·50 (-4·49 to -0·46) |


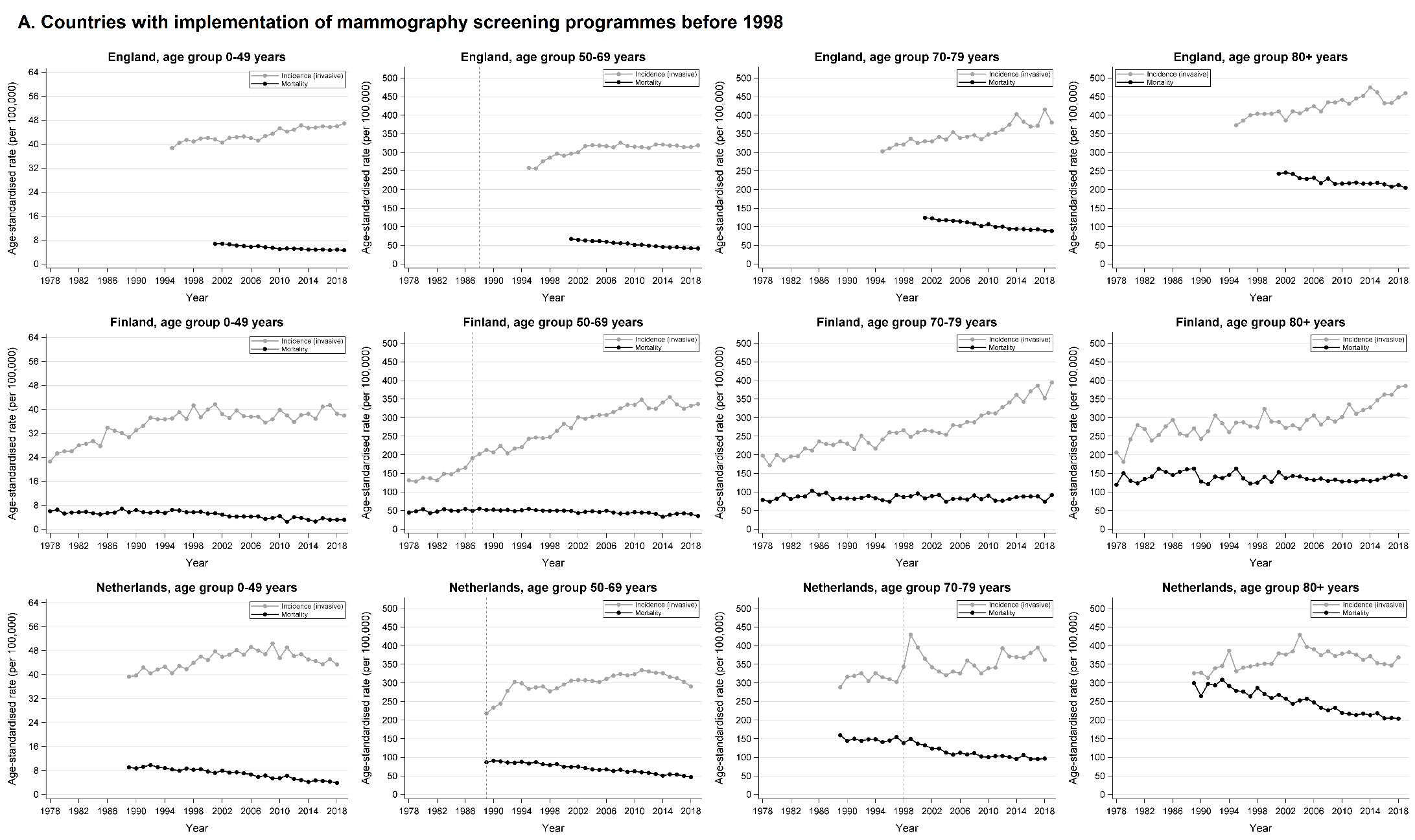


**Supplementary Fig. 1** Changes over time in age-standardised incidence of invasive breast cancer and mortality from breast cancer by country and age.

Vertical dashed lines represent years in which screening programmes were implemented.


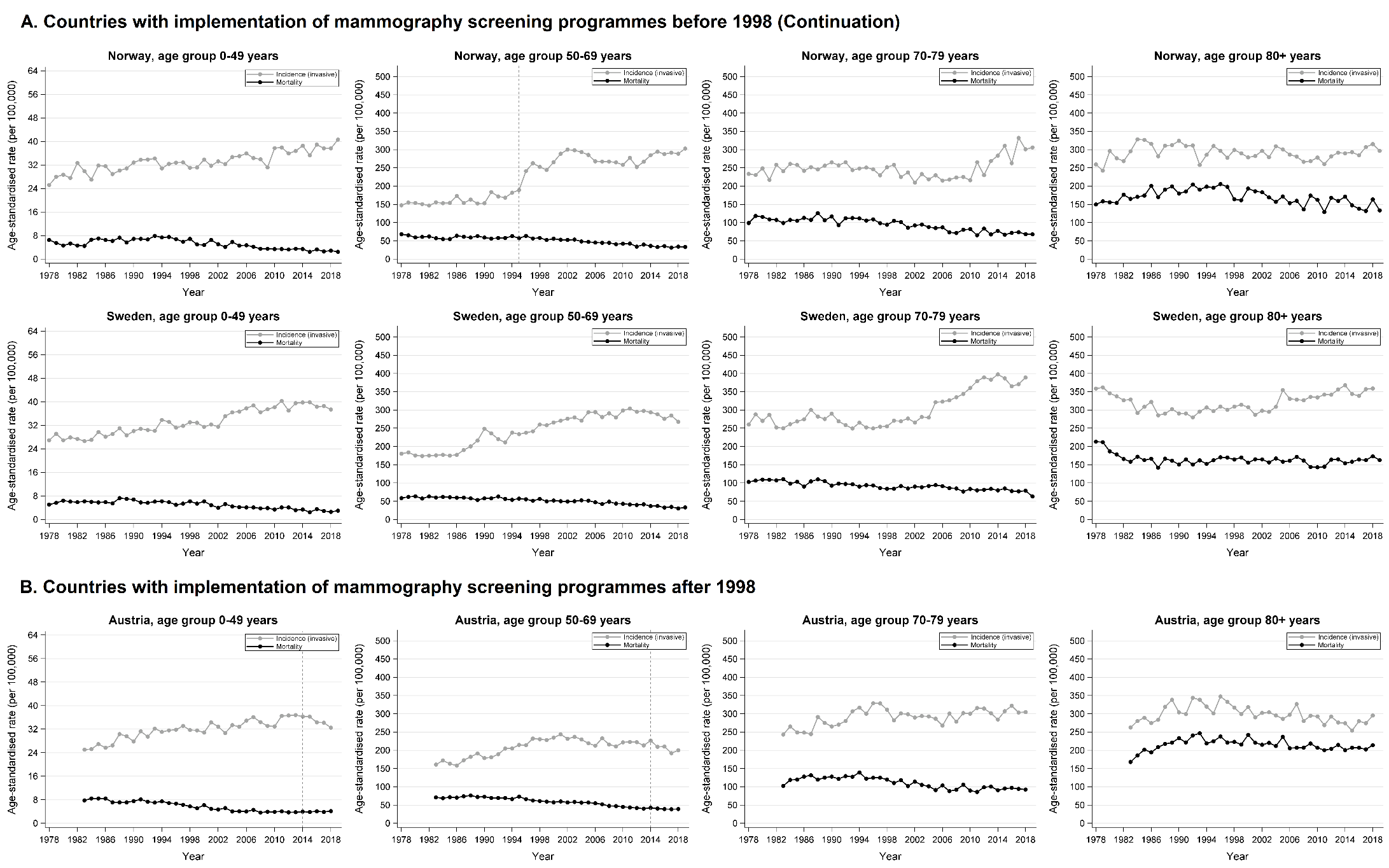


**Supplementary Fig. 1** Continued.


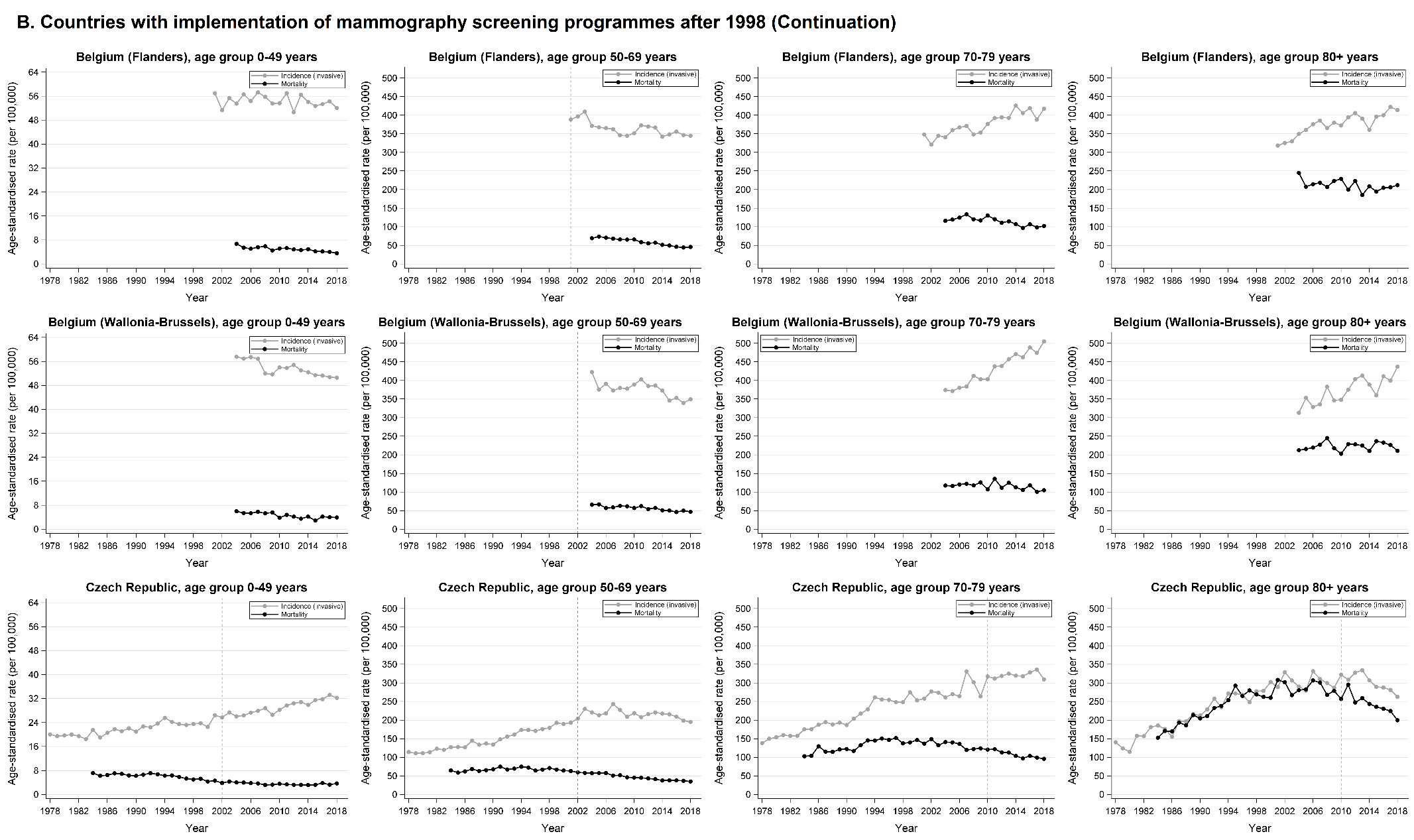


**Supplementary Fig. 1** Continued.


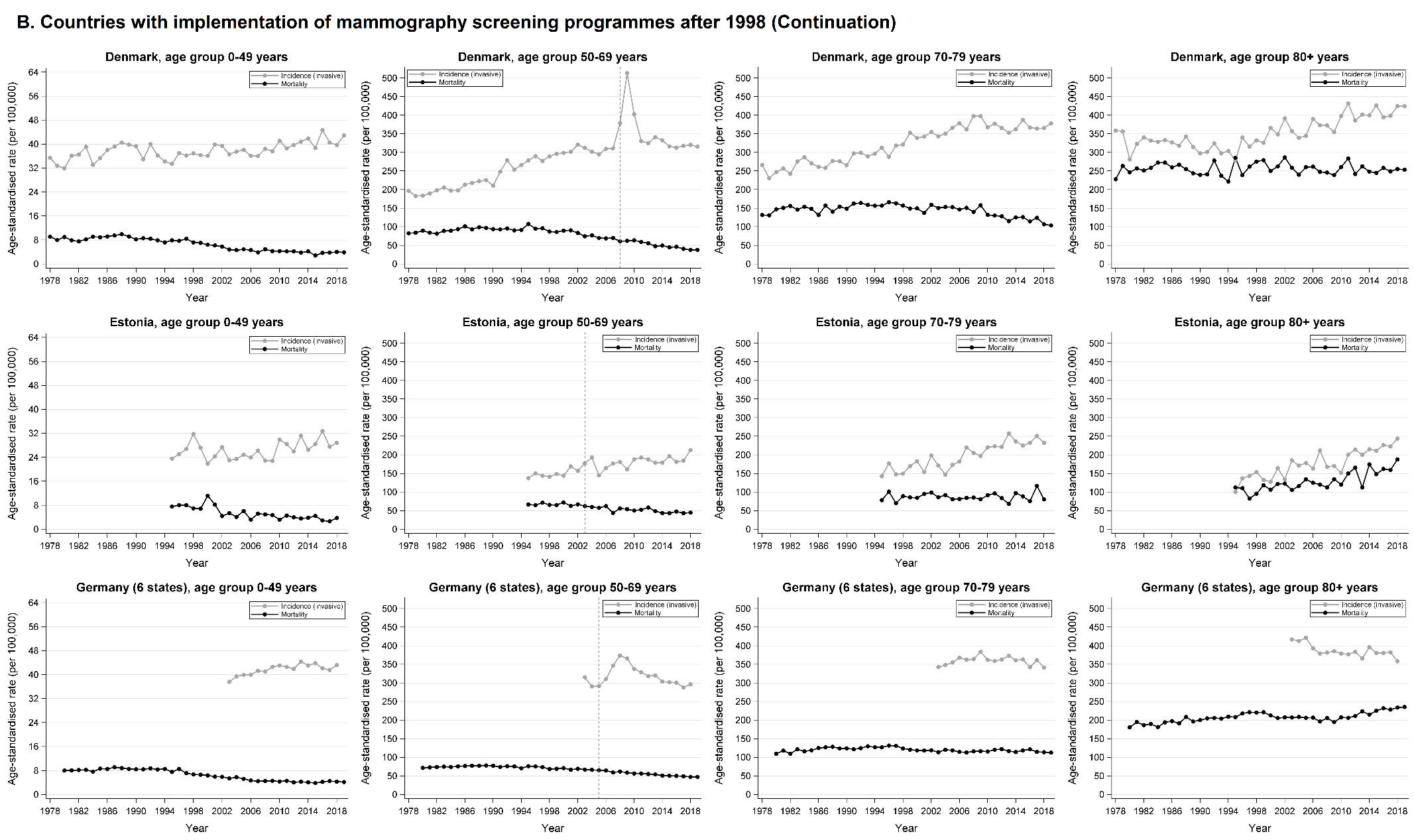


**Supplementary Fig. 1** Continued.


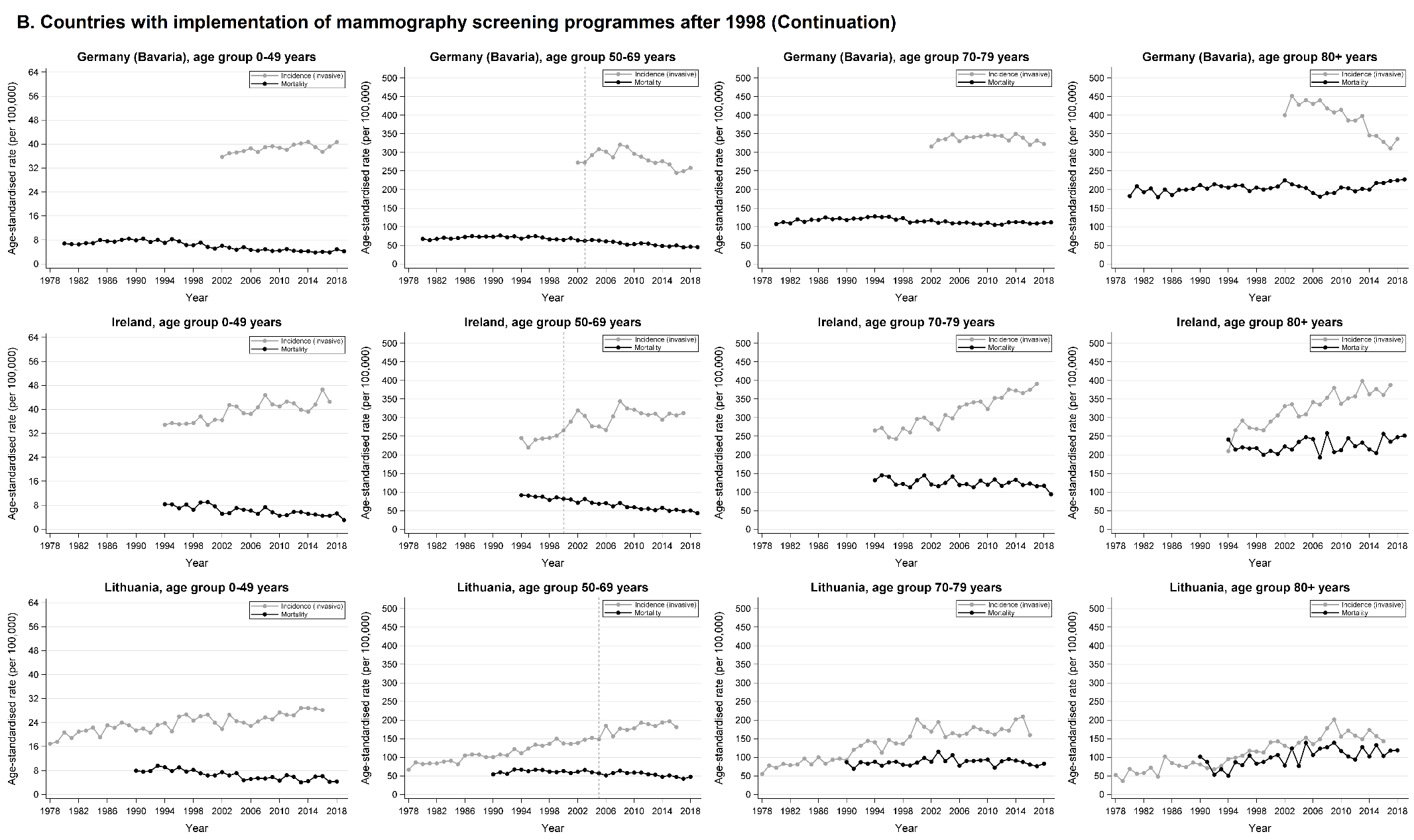


**Supplementary Fig. 1** Continued.


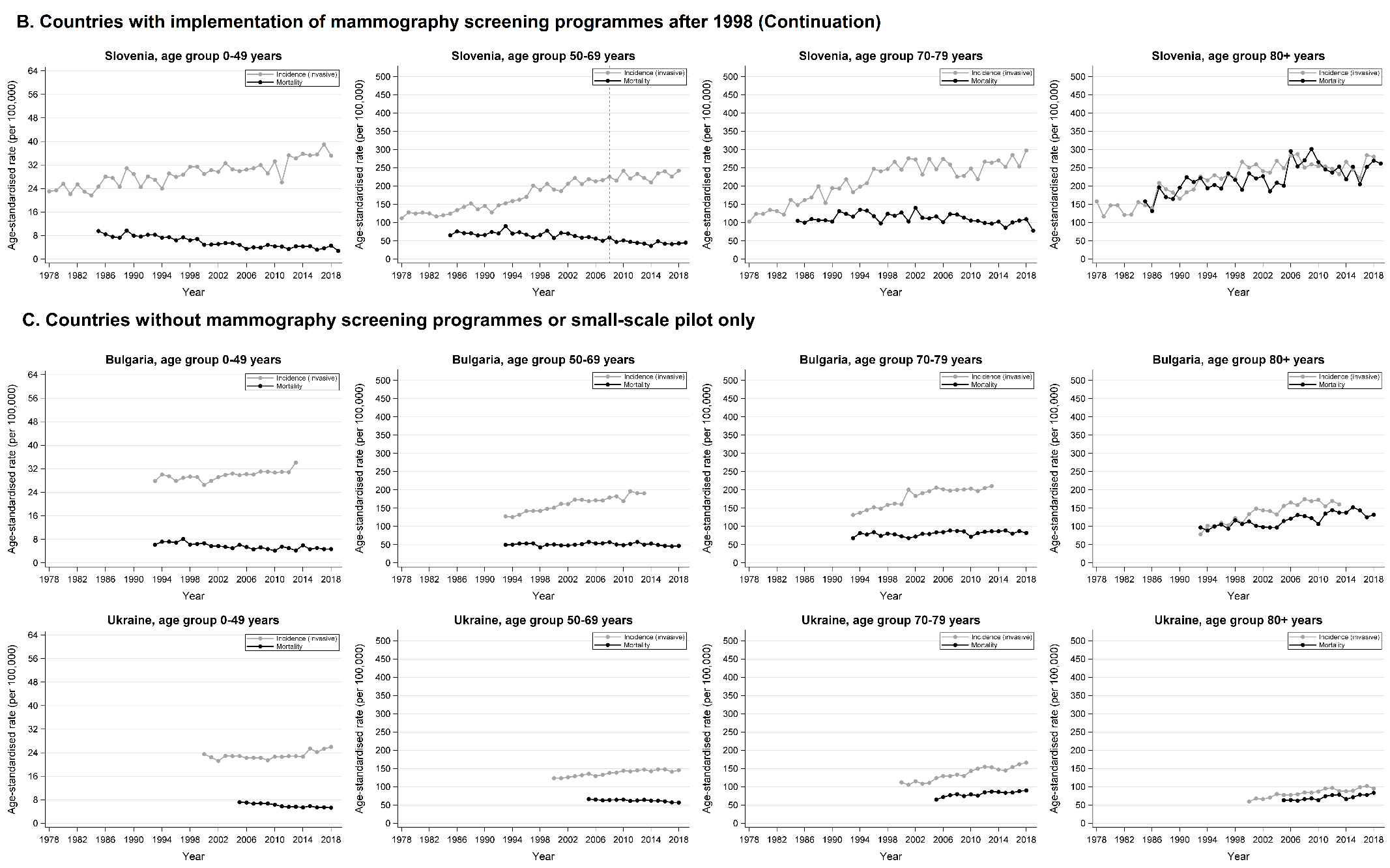


**Supplementary Fig. 1** Continued.


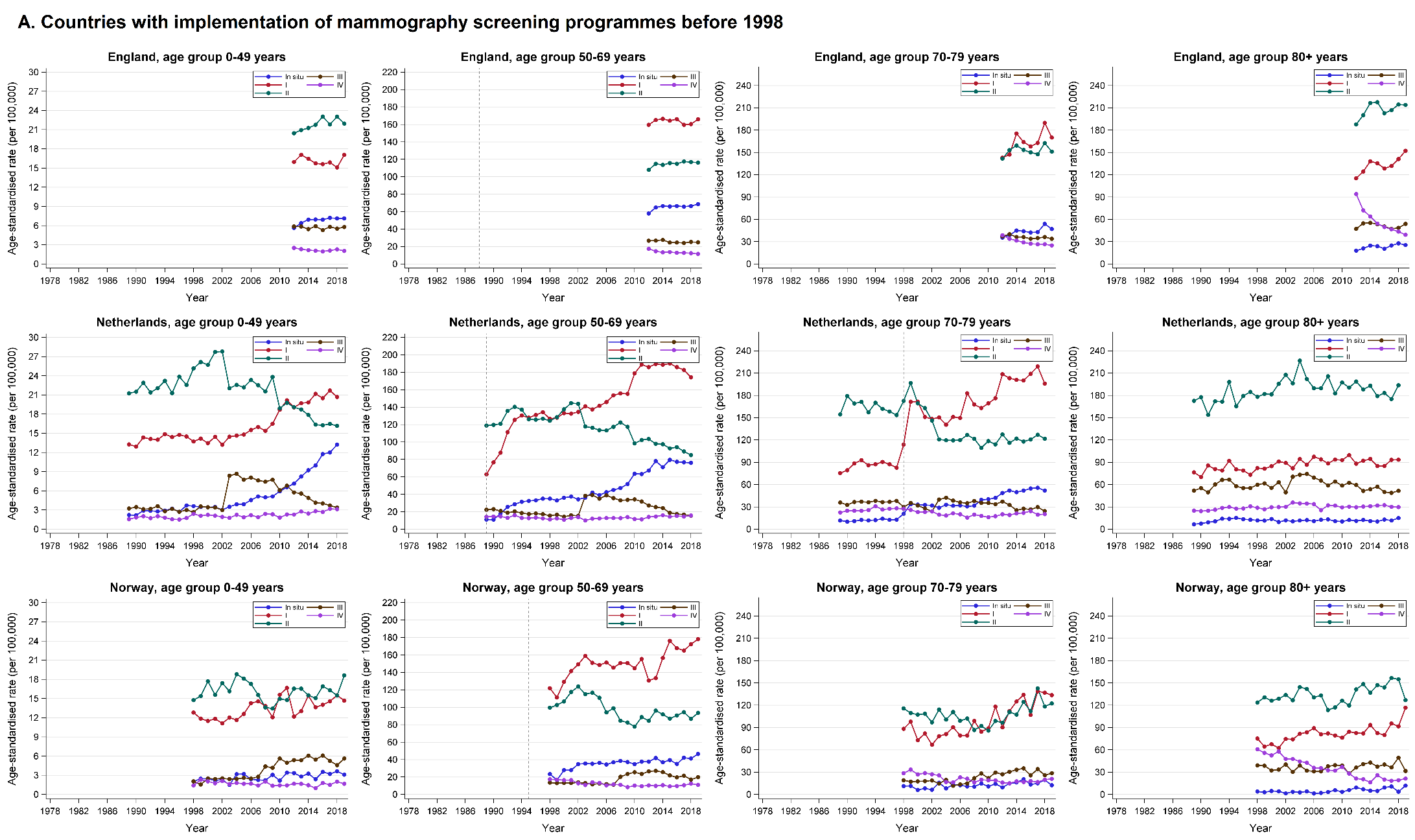


**Supplementary Fig. 2** Changes over time in age-standardised incidence of in situ, stage I, stage II, stage III, and stage IV breast cancer by country and age.

Vertical dashed lines represent years in which screening programmes were implemented.


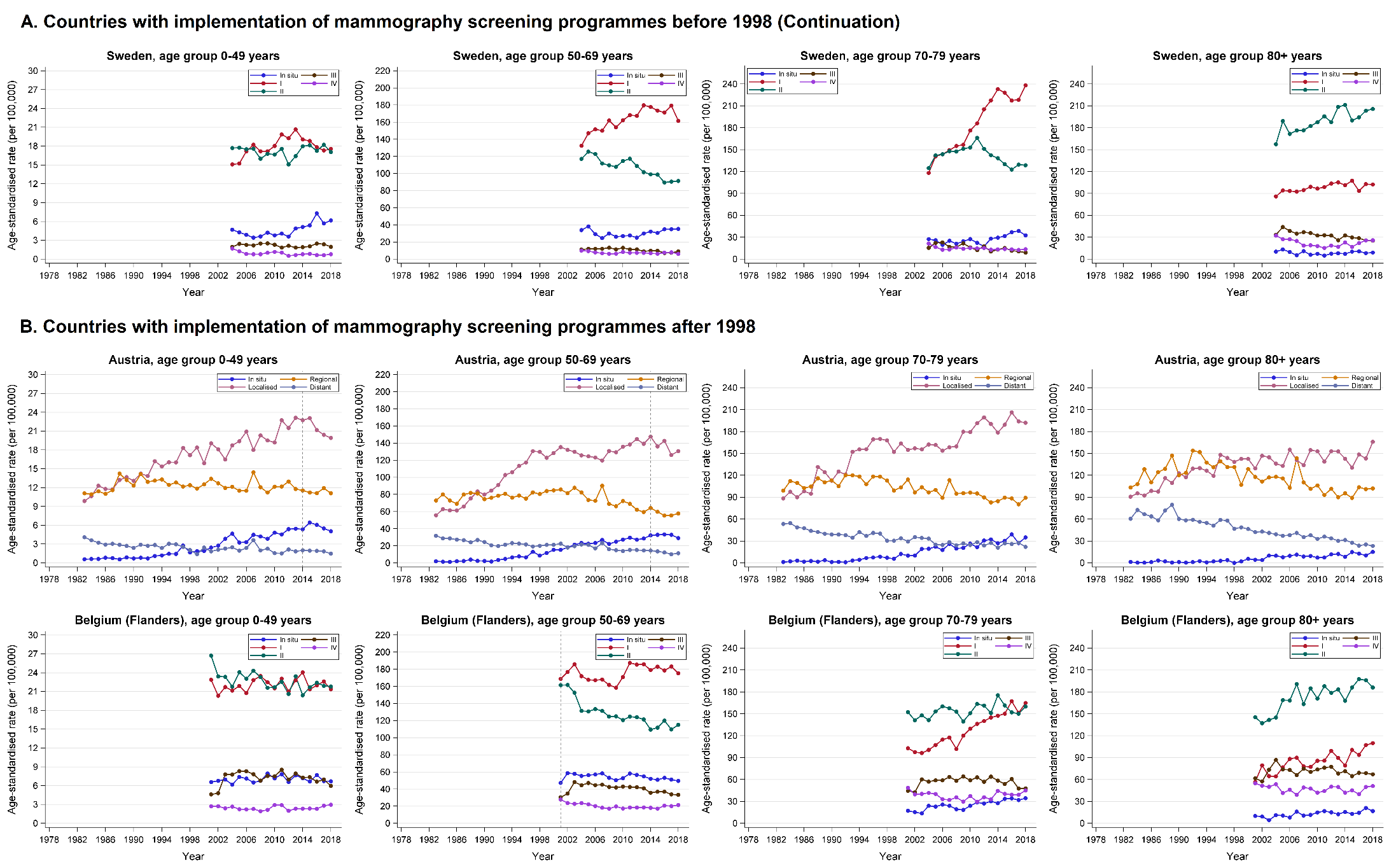


**Supplementary Fig. 2** Continued.


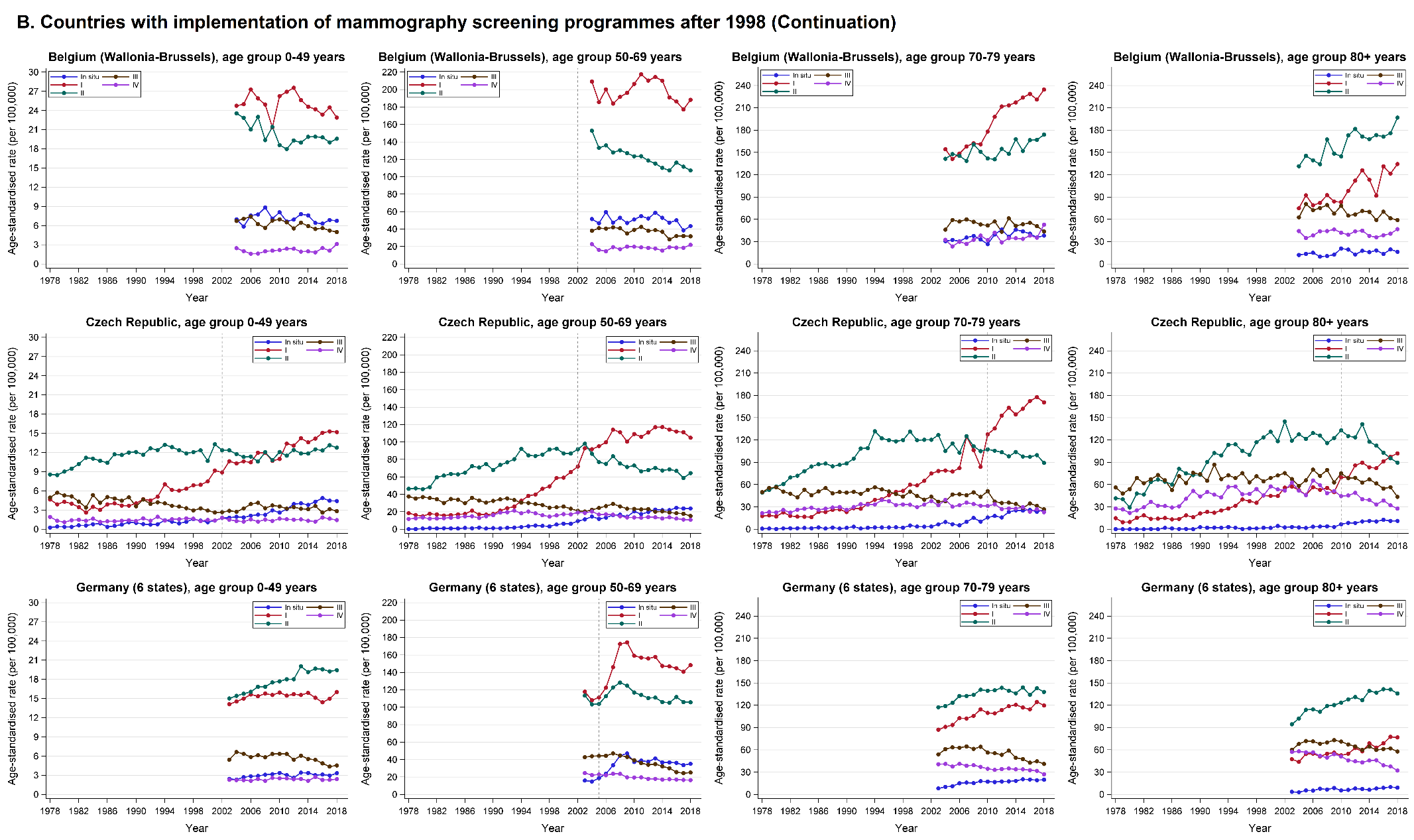


**Supplementary Fig. 2** Continued.


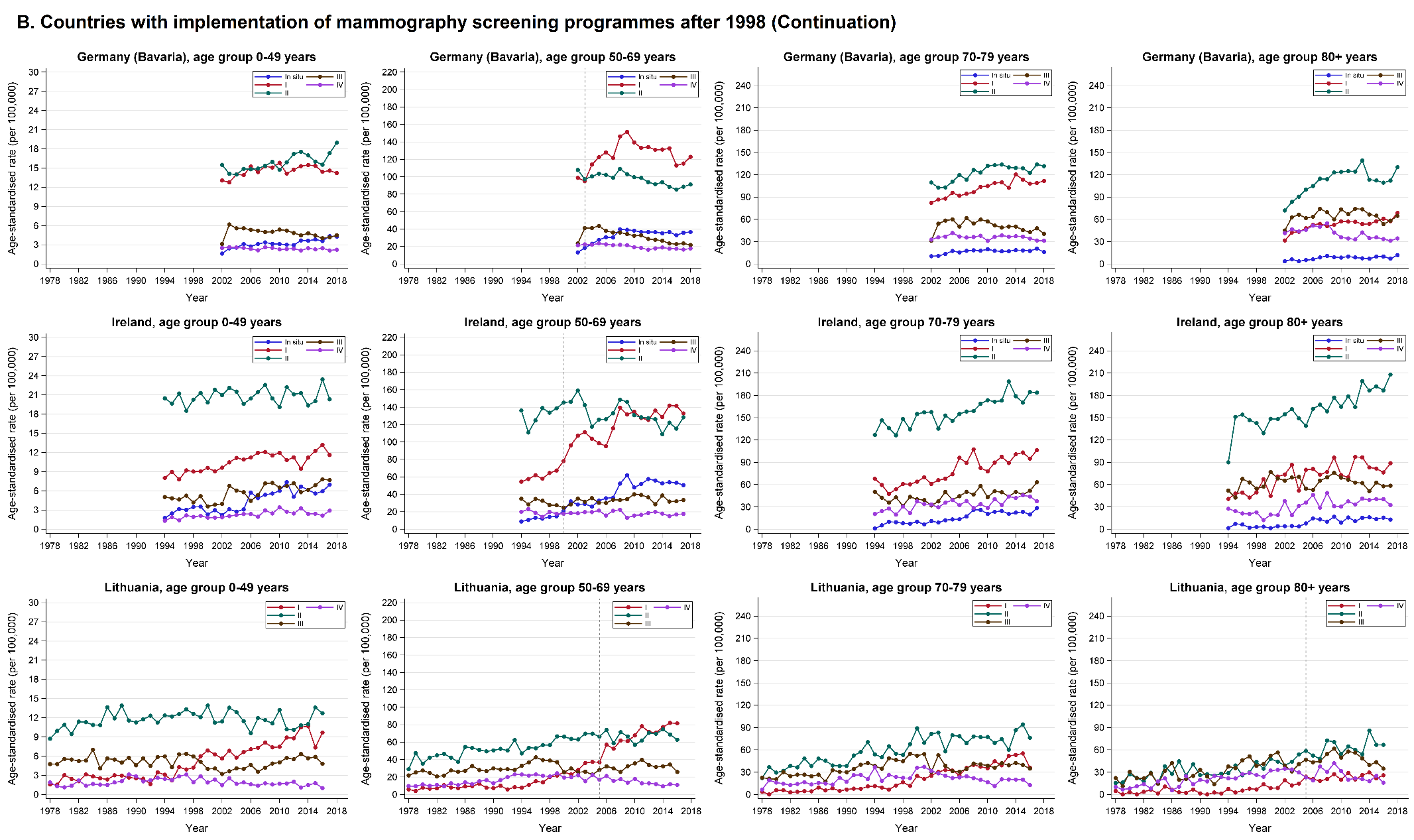


**Supplementary Fig. 2** Continued.

*
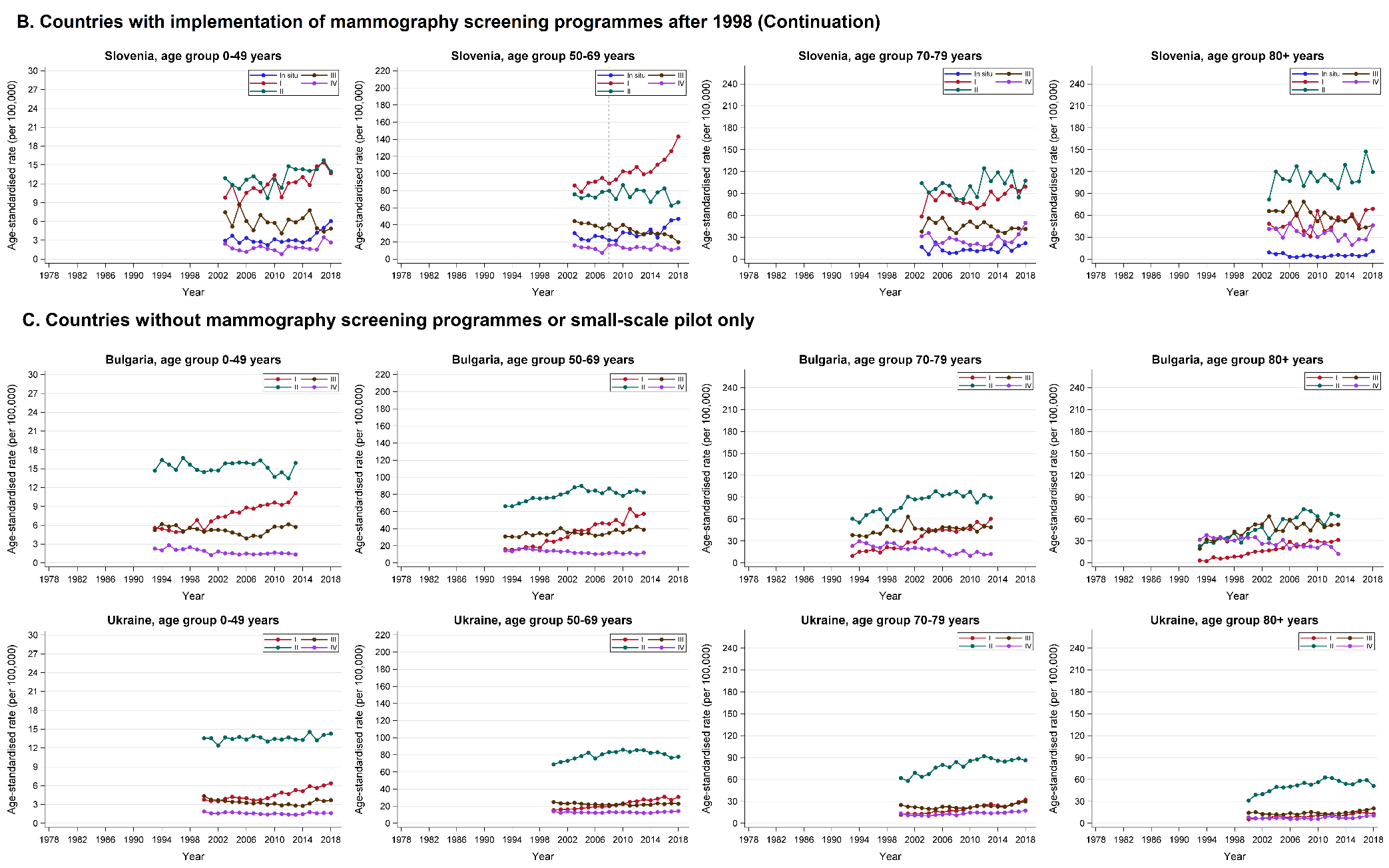
*

**Supplementary Fig. 2** Continued.

**Supplementary reference**

1. International Agency for Research on Cancer, International Association of Cancer Registries. International rules for multiple primary cancers (ICD­O third edition). (Lyon, 2004).
2. van Buuren S. & Groothuis-Oudshoorn K. mice: Multivariate Imputation by Chained Equations in R. J Stat Softw 2011; 45: 1 - 67.
3. US National Cancer Institute. Joinpoint Regression Program, Version 4.7.0.0; Statistical Methodology and Applications Branch, Surveillance Research Program.
4. Cardoso R, Hoffmeister M, & Brenner, H. Breast cancer screening programmes and self-reported mammography use in European countries. *Int J Cancer* 2023; **152**: 2512-27.
